# β-Thalassemia Patients Revealed a Significant Change of Untargeted Metabolites in Comparison to Healthy Individuals

**DOI:** 10.1038/srep42249

**Published:** 2017-02-13

**Authors:** Syed Ghulam Musharraf, Ayesha Iqbal, Saqib Hussain Ansari, Sadia Parveen, Ishtiaq Ahmad Khan, Amna Jabbar Siddiqui

**Affiliations:** 1 H.E.J. Research Institute of Chemistry, International Center for Chemical and Biological Sciences, University of Karachi, Karachi-75270, Pakistan; 2 Dr. Panjwani Center for Molecular Medicine and Drug Research, International Center for Chemical and Biological Sciences, University of Karachi, Karachi-75270, Pakistan; 3 Department of Pediatric Hematology & Molecular Medicine, National Institute of Blood Diseases and Bone Marrow Transplantation, Karachi-75300, Pakistan

## Abstract

β-Thalassemia is one of the most prevalent forms of congenital blood disorders characterized by reduced hemoglobin levels with severe complications, affecting all dimensions of life. The mechanisms underlying the phenotypic heterogeneity of β-thalassemia are still poorly understood. We aimed to work over metabolite biomarkers to improve mechanistic understanding of phenotypic heterogeneity and hence better management of disorder at different levels. Untargeted serum metabolites were analyzed after protein precipitation and SPE (solid phase extraction) from 100 β-thalassemia patients and 61 healthy controls using GC-MS. 40 metabolites were identified having a significance difference between these two groups at probability of 0.05 and fold change >1.5. Out of these 40 metabolites, 17 were up-regulated while 23 were down-regulated. PCA and PLS-DA model was also created that revealed a fine separation with a sensitivity of 70% and specificity of 100% on external validation of samples. Metabolic pathway analysis revealed alteration in multiple pathways including glycolysis, pyruvate, propanoate, glycerophospholipid, galactose, fatty acid, starch and sucrose metabolism along with fatty acid elongation in mitochondria, glycerolipid, glyoxylate and dicarboxylate metabolism pointing towards the shift of metabolism in β-thalassemia patients in comparison to healthy individuals.

β-Thalassemia is a common congenital haematological disorder which is characterized by dysregulation in the synthesis of the β-globin chain, one of the major constituents of adult haemoglobin (HbA)^1^. It is anticipated that annually 70,000 children are born with various types of thalassemia, and majority of these births are affected by severe forms of β-thalassemia^2^,^3^. Hundreds of mutations in the β-globin gene and/or regulatory elements associated with the β-globin gene are known to be the cause of this genetic haemoglobinopathy^4^. While in Pakistan, the most common mutation responsible for causing this blood disease is IVS1-5^5^.

In β-thalassemia, there is an imbalance in α/β-globin ratio and excessive α-globins possibly causes oxidative damage to membrane lipids and proteins of red cell in the form of irreversible hemichromes and also increases intracellular calcium, causing the significant increase in destruction of RBCs and ultimately anaemia^6^. Anaemia stimulates the erythropoietin production with subsequent intensive but ineffective expansion of the bone marrow (up 25 to 30 times normal), which sequentially causes the typical described bone deformities. Prolonged and severe anaemia and increased erythropoietic activity also result in hepatosplenomegaly, extramedullary erythropoiesis, iron induced dysfunctions of various organs, thrombosis, diabetes, severe infection, and growth retardation^7^.

Revealing alteration of metabolites in the course of a disease in body fluids and tissues is an emerging application in the field of biomedical research as this area has the possibility to act as an effective tool for predicting disease phenotype at early stage, and also predicting response of a treatment and survival^8^. In recent times, metabolomics has been used as potential biomarkers in organ transplantation and immunosuppressant toxicity^9^, assessing pathogenesis of lung diseases^10^,^11^, toxicology^12^, drug discovery and precision medicine^13^ and cancer biology^14^,^15^,^16^.

Many techniques have been used for screening and diagnosis of haemoglobin variants and thalassemia^17^. Determination of the genetic makeup of the person in question and characterization of human blood using complete blood cell count (CBC) are the most reliable methods for diagnosis of thalassemia. Still there is a limitation in the analysis of data due to a large number of possible candidate characteristics and various types of thalassemia and thalassemia trait^18^. Moreover, using such methods, there would be no information about alterations in the patterns of metabolites present in the biological materials that can give valuable phenotypic information and mechanistic insight into the biochemistry of disease processes and related abnormalities. Limited studies for markers identification in the blood or urine of β-thalassemia patients have been done which include the analysis of haemoglobin variants to diagnose thalassemia^19^, marker for lipid peroxidation-induced DNA damage^20^, plasma substance P and soluble P-selectin as biomarkers of β-thalassemia induced hypercoagulability^21^, adipocytokines related to haemolytic and inflammatory biomarkers^22^, biomarkers of iron and oxidant-antioxidant homeostasis^23^,^24^, nuclear magnetic resonance-based screening of thalassemia with quantification of some haematological parameters^25^ and quantification of the free α-Hb^26^. Various studies have been done showing that metabolic disorders are common in patients with β-thalassemia^27^,^28^ but to date, there is a lack of metabolomics based biomarkers that may play role in diagnosing the phenotype of β-thalassemia and convey prognostic approach with various management possibilities^26^.

This study focuses on the untargeted metabolomic analysis of β-thalassemia to gain insights into the molecular and cellular pathogenesis to aid in understanding the pathophysiology of disease at molecular level and to identify biomarkers/biomarkers pattern. Various powerful data mining and statistical bioinformatics methods were used for identifying, prioritizing and classifying robust and generalizable biomarkers with high discriminatory ability. No paper has been published so far regarding the profiling and identification of serum metabolomics of β-thalassemia patients. It is expected that findings of this study will strengthen the knowledge of molecular mechanisms involved in β-thalassemia that will ultimately help in the identification of the characteristic molecule(s) as well as improvement of possible therapy of the disease.

## Material and Method

### Patient’s Selection

The selection of patients for present study was carried out in National Institute of Blood Disease and Bone Marrow Transplantation (NIBD), Karachi, Pakistan after the ethical approval of the Institutional Review Board (IRB), ethic committee of hospital while the experimental protocols were approved by the primary research institute (International Center for Chemical and Biological Sciences, ICCBS). This study included 100 cases of β-thalassemia with following inclusion criteria: Patients have been registered at NIBD, diagnosed as case of β-thalassemia major (defined as Hb <7 g/dL, high HbF, absent or very low HbA, and more than 8 transfusions a year), sampling was carried out prior to blood transfusion if patient requires blood transfusion, and in 4–8 hours fasting condition. Exclusion criteria included: Patients having any evidence of other chronic illnesses unrelated to thalassemia and unwillingness to enrol in the study. Healthy volunteers were also recruited at Dr. Panjwani Center for Molecular Medicine and Drug Research (PCMD) for this study, basic details of patients and healthy controls are mentioned in supplementary information (Table S1). Written informed consent was obtained from all the participants of this study including control and patients. A thorough questionnaire consisting of questions for information required for study was also filled from all the patients.

### Sample Collection

Sample collection was carried out in accordance with relevant guidelines and regulations. Blood was collected from participants after 8 hours fasting but for infants and toddlers sample was collected when they felt need for food. About 5 cc of blood was drawn from participants by venepuncture and collected in gel-based BD vacutainer tubes (BD Franklin Lakes NJ, USA, REF: 367381), interior coated with silicone for clot activation. Serum was separated by centrifugation at 2000 rpm for 10 minutes at 4 °C. And then serum was aliquoted and stored immediately at −80 °C freezer till further processing of sample.

### Reagents and Solvents

Analytical grade solvents were used for GC-MS analysis. Reagents and solvents included methanol, hexane (Tedia, Tediaway, Fairfield, USA), myristic-d_27_ acid, *N,O*-bis(trimethylsilyl) trifluoroacetamide (BSTFA) with trimethylchlorosilane (TMCS) (Sigma-Aldrich, St. Louis, Missouri, USA), methoxylamine hydrochloric (Acros Organic, New Jersey, USA), Pyridine (Lab-Scan, Bangkok, Thailand) and Mill-Q/deionized water (Millipore, Billerica, MA, USA).

### Sample Preparation and Derivatization

Protocol used for preparation of samples in this study for profiling of metabolites has been reported previously in detail and it include methods that were performed in accordance with the relevant guidelines and regulations^29^. In short, first proteins were precipitated by adding 800 μL of chilled methanol in 100 μL serum containing 20 μL of myristic acid (2 mg/mL) as internal standard. Supernatant was subjected to solid phase extraction using a 96 well plate (Strata C18-E, 55 μm pore size, 70 Å particle, 100 mg sorbent/1 mL Phenomenex, USA) under vacuum (AHC-7502, Phenomenex, USA). After sample loading, the solid phase was washed with 300 μL of water and metabolites were eluted with 600 μL methanol and collected in a 96 well collection plate. The eluent was finally dried in vaccum at room temperature. The dried extract was stored at 4 °C until analysis. Derivatization of dried samples was carried out by addition of 50 μL methoxylamine hydrochloride (15 μg/μL in pyridine) followed by addition of 50 μL BSTFA with 1% trimethylchlorosilane for formation of trimethylsilyl derivatives. Then sample was centrifuged and analyzed on GC/MS.

### GC-MS Analysis

GC-MS analysis of derivatized samples was executed as mentioned previously with minor modifications in the GC method^30^. Analysis was carried out on 7890 A GC (Agilent technologies, USA), fitted with a GC auto sampler 120 (PAL LHX-AG12–Agilent Technology) autosampler and coupled to Agilent 7000 Triple Quad system (Agilent Technologies, USA). A fused-silica capillary GC column, HP-5MS 30 m x 0.25 mm ID (Agilent J&W Scientific, Folsom, CA, USA), chemically bonded with a 95% dimethylpolysiloxane 5% diphenyl cross-linked stationary phase (0.25 mm film thickness) was used. The serum sample was injected in the splitless mode using helium as carrier gas. Initially the oven temperature was fixed at 50 °C for 1 min then temperature was raised in three steps. In first step temperature raised at a rate of 10 °C per minute to 80 °C for 3 min then again 10 °C per min to 180 °C for 3 min and in final step 15 °C per min raise to 300 °C for 5 min. After maintaining the temperature at 300 °C for five minutes, it was further increased to 305 °C for one minute which referred as post run. Retention time was locked to the internal standard at 20.070 min. Electron impact ionization (EI) was used as an ionization source for the GC/MS analysis at 70 eV. Data acquisition was done in full scan mode from 50–650 *m/z* in 0.5 seconds scan time. A blank was run between samples to remove contamination. Mass calibration was done with perfluorotributylamine (PFTBA).

### GC-MS Data Preprocessing and Statistical Analysis

Agilent Mass Hunter Qualitative Analysis software (version B.04.00) was used for data processing. Peak integration and deconvolution parameters have been previously reported^29^,^30^. Mass spectra of the peaks were compared with NIST mass spectral (Wiley registry NIST 11) library leading to presumptive identification of metabolites with a ≥ 70% similarity index. The GC-MS spectra were uploaded on Mass Profiler Professional (MPP) software 12.5. Filtering of the data involved using all available data and minimum absolute abundance of 5,000 counts with 3 number of ions minimum. Match factor 0.3, retention time tolerance 0.05 and delta MZ (low resolution) 0.2 were set as alignment parameters. External scalar was used for normalization of data. Z transform was selected as base lining option treating all the compounds equally irrespective of their intensity. A total of 711 compounds were detected in the entire samples after alignment of data. Statistical significance analysis was done using student T-test unpaired for healthy versus β-thalassemia patients of fold change (FC) 1.5. A PLSDA model was built for healthy versus β-thalassemia patients using auto scaling, N fold validation type, three number of folds and ten number of repeats. Blind samples (n = 20) were also run for external validation. Sensitivity and specificity of the constructed model were also measured.

## Results

**Figure 1:**
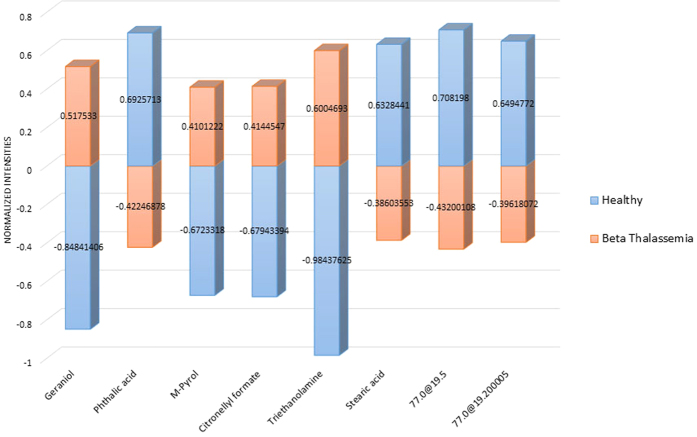
Eight metabolites at FC >2, out of which 4 are down-regulated (bar below the baseline of 0) while 4 are up-regulated (bar above the baseline of 0) in β-thalassemia patients as compared to healthy group.

Metabolite profiling of total 161 serum samples including healthy volunteers (n = 61) and β-thalassemia patients (n = 100) were analysed by using GC-EI-MS. After performing GC-MS analysis as described above, identification of metabolites was carried out using Agilent Mass Hunter Qualitative Analysis software and NIST library. Statistical and multivariate data investigation i.e. Heatmap, PCA plot and PLSDA plot was carried out using MPP software in order to identify comparative and statistically significant metabolites between healthy and β-thalassemia samples. Significant testing and fold change was carried out on total of 711 entities found in this experiment. Student’s T-test unpaired, asymptomatic p-value computation and multiple testing correction by Benjamini Hochberg FDR was applied. A list of 40 compounds was generated at probability of 0.05 and fold change >1.5. Out of these 40 metabolites, 17 metabolites were up-regulated and 23 were down-regulated in β-thalassemia patients in comparison to healthy controls as listed in Tables 1 and 2, respectively with their CAS registry numbers. Among these 40 metabolites, 8 were showing a fold change of 2 among disease and healthy group as shown in Fig. 1. Twenty one out of forty low molecular weight metabolites; geraniol, palmitic acid, α-glyceryl palmitate, lactic acid, α-glyceryl stearate, M-pyrol, citronellyl formate, sucrose, triethanolamine, 5-ethyl-5-methyldecane, 2,3-dimethyl-2,3-butane-diol, boric acid, phosphoric acid, hexadecane, methylbis(phenylmethyl)benzene, dodecane, 4,6-dimethyl, phthalic acid, glycerol, stearic acid, n-pentatriacontane and ethylene glycol; were putatively determined by comparing the mass spectra of the peaks with those available in the NIST mass spectral (Wiley registry NIST 11) library at ≥ 70% similarity index while the remaining were not identified at this similarity index. The identified compounds are shown with their name while unidentified with their base peak and retention time in Tables 1 and 2. The EI/MS spectra of remaining nineteen unidentified compounds are shown in supplementary information (Figure S1). Principal component analysis (PCA) was also carried out on our data to make sure that the difference in metabolic pattern is due to difference in health status and not attributable to age or weight. We found that samples were not separating on the basis of age or body mass shown in supplementary information (Figures S2 and S3) therefore excluding these confounders.

**Figure 2:**
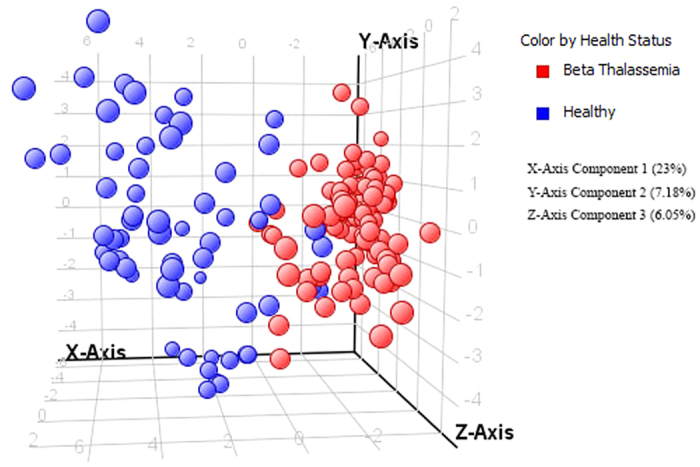
PCA score plot of healthy and β-thalassemia patients based on forty (40) significantly differentiated metabolites i.e. having a fold change >1.5. Variance on X, Y and Z axis is also shown within brackets.

**Figure 3:**
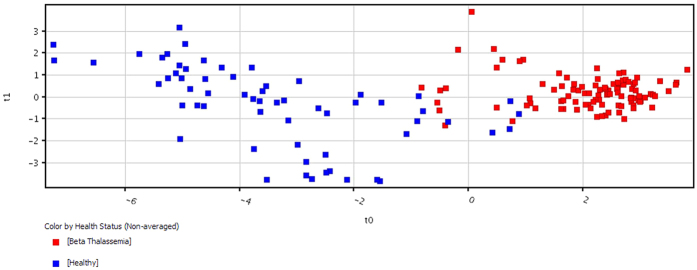
PLSDA scores scatter plots discerning among healthy controls and β-thalassemia patients based on forty (40) significantly differentiated metabolites i.e. having a fold change >1.5.

PCA was carried out and a model was generated which revealed a vibrant and noteworthy difference between the non-averaged healthy samples and β-thalassemia samples. The PCA scores are shown in Fig. 2 in which each sample is denoted by a single point. The 50% cumulative variance of samples was observed at component 6 and variance of first three components on X, Y and Z axis are found to be 23%, 7.18% and 6.05% respectively. So this indicates that there are many factors responsible for discriminating metabolites between the healthy and disease group. A prediction model of healthy versus disease group was built by multivariate data analysis that include all analysed samples i.e. 100 β-thalassemia and 61 healthy samples and on the basis of forty metabolites having a statistically important difference in expression between these two clusters. Samples were classified into discrete classes also by supervised Partial Least Square Discriminant Analysis (PLSDA). Two parts of the input data were randomly assigned to the training set and remaining into the testing set. Auto-scaling was applied which involves subtracting the variable mean from each variable (data column) and dividing each by its standard deviation. This process was repeated ten times, each time using a different part for testing thus using each row once in training and testing generate a Confusion Matrix, which gives accuracy of prediction for each class. Plots obtained by PLS-DA scores are shown in Fig. 3 exposing an unblemished separation trend between the two sets of our experiment. Sensitivity of the constructed model was calculated from the proportion of β-thalassemia samples that were predicted correctly and referred as true positives, while specificity was determined from the proportion of control samples which were correctly predicted and these are stated as true negatives. Sensitivity of our built model was found to be 92.0% and specificity was 95.0%, respectively, while the overall accuracy of the model was 93.1% as mentioned in Table 3. The predictive capacity (i.e. sensitivity and specificity) of the model was measured also by external validation using 20 serum samples consisting of 10 samples each from healthy controls and β-thalassemia patients. But these samples were decoded prior to preparation and analysis by GC-MS therefore these were an independent or blind test set of samples. External validation correctly predicted the presence of β-thalassemia in 7 out of 10 patients and healthy controls in 10 out of 10 patients resulting in a sensitivity of 70% and specificity of 100%. Sample prediction reports are shown in Figure S4 of supplementary information.

To identify metabolic pathways those are disturbed in β-thalassemia we used web based software MetaboAnalyst 3.0 (www.metaboanalyst.ca/) in which previously mentioned list of identified metabolites was entered. On the basis of several databases such as KEGG (Kyoto Encyclopedia of Genes and Genomes) (http://www.genome.jp/kegg/)^31^,^32^ and HMDB (Human Metabolome Database) (http://www.hmdb.ca/) this online software helps in identifying the pathways with significant alterations. The summaries of pathway analysis created on the basis of hypergeometric test and relative-betweeness centrality in pathway topology analysis by this program using up-regulated and down-regulated metabolites are shown in Figures S5 and S6, respectively. The images of distinguishing identified pathways are provided in supplementary data i.e. Figure S7.

## Discussion

The innovative omics technology has opened up exciting opportunity for screening and identification of novel biomarker that can acts as an indicator for the physiological alteration of body. Evolving technologies of metabolomics profiling hold potential for lighting biology and human diseases. Metabolites have a wide range of functional groups are present from volatile alcohols, ketones, amines, organic acids to complex lipids, carbohydrates and other secondary metabolites. β-thalassemia is one of the very frequent and extremely disabling genetic disease. Different pathological and environmental stresses change expression level of certain genes and hence concentrations of metabolites of corresponding pathways. Therefore, we aimed to determine these changes in metabolome of β-thalassemia patients for disease prognosis and to understand unclear pathophysiological mechanisms of thalassemia, as field of metabolomics has proven itself a promising technique in understanding pathophysiology of many other various diseases also including genetic diseases such as sickle cell anaemia^33^,^34^,^35^,^36^. However, our results are limited by the fact that the type of mutation was not known in patients.

**Figure 4:**
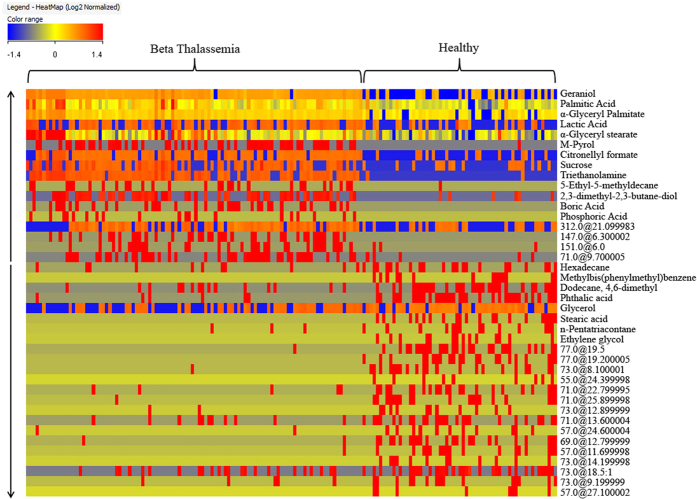
Image of heatmap using non-average samples with normalized intensities of 40 significant metabolites that showed significant difference among healthy & disease group. The arrows indicate the up-regulation and down-regulation of metabolites.

The comparison of serum metabolites between β-thalassemia patients and normal subjects revealed evident alterations of metabolites in the disease group. The close image of heat map using non-average samples with normalized intensities of forty (40) significant metabolites are shown in Fig. 4 in which the identified metabolites are stated by their name while unidentified with their base peak and retention time. From this heat map it is quite clear that β-thalassemia metabolite profile is totally different from the control group and it can also be observed that the concentration of some of the metabolites is increased while of some is decreased. This change in metabolite profile indicates that in β-thalassemia patients metabolism is shifted from the normal state and it is also reported in literature that metabolism is disturbed in this genetic disease^37^. Hence, knowledge related to these altered metabolites play an important role in understanding of disease progression at molecular level. Significance of the transformed metabolite profile in β-thalassemia can be described by referring it to the human metabolome database (HMDB)^38^,^39^,^40^.

### Up-regulated metabolites

Geraniol also named as rhodinol is a monoterpenoid and an alcohol which occurs in essential oils of several aromatic plants. Its bio-functions include cell signalling, storage and source of fuel or energy and integrity of membrane. It possess anti-cancer, antimicrobial, anti-oxidant, anti-inflammatory and some vascular effects^41^, therefore it is possible that geraniol is increased as a result of oxidative stress, inflammation and decreased RBCs membrane integrity in β-thalassemia. Palmitic acid or hexadecanoic acid is one of the most common saturated fatty acids found in animals, a saturated fatty acid found in fats, waxes and body lipids. It is involved in various metabolic pathways in body and its altered levels are also reported in colorectal cancer, breast cancer, eosinophilic esophagitis and gastroesophageal reflux disease. Palmitic acid plays important functions other than providing energy^42^. One of their functions is to induce apoptosis, so their high levels may be responsible for early degradation of RBCs in β-thalassemia patients. α-Glyceryl palmitate and α-glyceryl stearate are forms of fatty acid, both are one fatty acid chain containing glycerides, covalently bonded to a glycerol molecule through an ester linkage. Both of these are source of energy as well as required also in maintaining stability of membrane so its increase levels can be due to more destruction of red cells. Lactic acid is a crucial metabolite which is involved in various biochemical processes and its production take place due to extreme activity in muscles. It is a component of various metabolic pathways such as cysteine, propanoate and pyruvate metabolism. As it is a product of anaerobic glycolysis therefore its enhancement in β-thalassemia can be predicted as these patients have low Hb levels which cause less supply of oxygen to tissues and leading to more anaerobic glycolysis. The second reason for more lactic acid levels, that it may be due to poor hepatic function a consequence of iron overload in these patients as abnormal concentrations of lactic acid are found in hepatic biliary malignancies in addition to other cancers. M-pyrol is a product of GABA (γ-aminobutyric acid) a neurotransmitter, its altered levels are seen in bladder infection and urinary tract infections are common in β-thalassemia because of predisposing factors of such as splenectomy, iron overload, anaemia, and granulocyte dysfunctions^43^. Sucrose is a non-reducing disaccharide of glucose and fructose and is linked to various metabolic pathways of glucose and other sugars. As it is broken down into its constituents fructose and glucose and its increase in blood indicates metabolic syndrome, mostly in β-thalassemia patients glucose homeostasis is abnormal this may result in its elevation^28^. Citronellyl formate and triethanolamine are metabolites found in cytoplasm as well as extracellularly. Both of them are produced endogenously in addition to dietary source. Phosphoric acid is another important metabolite which we found to be up-regulated in the disease group. It is present in cytoplasm and it act as an osmolyte and enzyme cofactor in biological system. It is also involved in signalling and a list of metabolic pathways such as ammonia recycling, arginine, proline, cysteine, purine, pyruvate, inositol metabolism and various glucose metabolic pathways. Therefore, it can be stated that increase phosphoric acid levels are indicator that various metabolic pathways are up-regulated in this disease.

### Down-regulated metabolites

Hexadecane is a 16 carbon atoms chain that has been shown to exhibit anti-inflammatory, anti-bacterial, anti-oxidant and thermogenic functions. Because in β-thalassemia all these activities are increased so low levels of hexadecane can be co-related with its more utilization and less availability in serum freely. Phthalic acid is a toxin or pollutant found in blood and when found in tissues or biofluids, it arises from exposure to phthalate products. Phthalate is an environmental chemical of high public concern because reports of its potential risk to male reproductive health so it can be said that iron load is a major reason of infertility in these patients and not the environmental toxins^44^. Glycerol is a major component of phospholipids and triglycerides that can be converted into glucose by liver to fulfil energy requirements. It is a component of glycerolipid and glycerophospholipid metabolism. And its abnormal levels have been quantified and identified in various disorders^45^,^46^, so it is obvious that in β-thalassemia glycerol is more consumed for energy production due to metabolic stress. Octadecanoic acid or Stearic acid is a beneficial saturated fatty acid involved in mitochondrial beta-oxidation of long chain saturated fatty acids and plasmalogen synthesis. These both pathways mainly contribute to maintain dynamics of membrane and cell signalling so low levels of stearic acid in body further contribute to decrease strength of RBCs membrane and altered cell signalling. Ethylene glycol also known as polyethylene oxide (PEO) or polyoxyethylene (POE), depending on its molecular weight is an oligomer or polymer of ethylene oxide. It functions in biosystem as a nutrient, anesthetic, anti-microbial, laxative and radical scavenger and its low levels may further aggravate the oxidative stress and increase susceptibility to infections.

### Pathway Analysis

Pathways were produced from MetPA (Metabolomic Pathway Analysis) software that showed dysregulation in β-thalassemia patients (Table 4). Using MetPA identified metabolites were analysed that contains pathways from the KEGG metabolic pathways database and HMDB. Pathway enrichment with topology analysis, and an interactive visualization system is also used to find pathways that are most substantially altered under the conditions of particular experiment. In metabolic networks, more severe effects are produced due to changes in more “vital” locations on the pathway compared to variations occurring in bordering or comparatively isolated positions. In our analysis, we identified several pathways some were generated from list of metabolites that were up-regulated in β-thalassemia, including fatty acid elongation in mitochondria, glycolysis or gluconeogenesis, pyruvate, propanoate, glycerophospholipid, galactose, fatty acid biosynthesis and metabolism, starch and sucrose metabolism that may be amplified in these patients. While the metabolites that were down-regulated in β-thalassemia patients showed abruption in glycerolipid, galactose, glyoxylate and dicarboxylate metabolism and fatty acid biosynthesis. Metabolites involved in dysregulation of these pathways are palmitic acid, lactic acid, sucrose, triethanolamine, glycerol and ethylene glycol. The detail results of pathway analysis are shown in Table 4 illustrating all matched pathways according to p-values from pathway enrichment analysis and pathway impact values from pathway topology analysis.

The pathways with considerable impact include glycerolipid, pyruvate, galactose, starch/sucrose and fatty acid metabolism while metabolites blameable for these deviations are glycerol, lactic acid, sucrose and palmitic acid respectively. We also noted that two pathways are found in both lists of pathways altered either due to increase or decrease of metabolites. One of them is fatty acid biosynthesis so it can be assumed that increase in palmitic acid is compensated by body with decrease in stearic acid to sustain the regulation of fatty acid biosynthesis pathways. Other is galactose metabolism in which increase sucrose is responsible for this pathway alteration which is compensated by decrease in glycerol. But this compensation is not sufficient enough by body because decreased glycerol has no such significant impression on this pathway as compared to increased sucrose. Therefore, it can be anticipated that diabetes an important complication of β-thalassemia is linked to imbalanced and aggravated galactose metabolism.

## Conclusion

This study showed that genetic abnormalities in β-thalassemia also give rise to disturbance in metabolism of body that can be observed by alteration in serum metabolomic profile of β-thalassemia patients as compared to the profile of healthy group. Our research demonstrated that metabolite profiling by GC-EI-MS is a reproducible, sensitive and less invasive method that can be used for establishment of a profile distinguishing between β-thalassemia patients and healthy controls with a good sensitivity and specificity. A model was fabricated on forty significantly expressed metabolites precisely classifying β-thalassemia patients and healthy controls on external validation. In addition to this many important pathways are identified that were found to be impaired in β-thalassemia and may play role in disease progression. Moreover, our approach is the first to report differences in the serum metabolome between healthy and β-thalassemia patients, a molecular level understanding that can be used in improving treatment options for the sufferers as well as diagnosing phenotype of patients.

## Additional Information

**How to cite this article:** Musharraf, S. G. *et al*. β-Thalassemia Patients Revealed a Significant Change of Untargeted Metabolites in Comparison to Healthy Individuals. *Sci. Rep.* **7**, 42249; doi: 10.1038/srep42249 (2017).

**Publisher's note:** Springer Nature remains neutral with regard to jurisdictional claims in published maps and institutional affiliations.

## Supplementary Material

Supplementary Information

## Figures and Tables

**Table 1:** List of up-regulated metabolites in β-thalassemia patients in comparison to healthy controls.

S. No.	Compound (CAS No.)	Compound Found in No of Disease Samples/100	Compound Found in No of Healthy Samples/61	Retention Time	p (Corr) ([Disease] Vs [Healthy]) :Normalized	Log FC (abs) ([Disease] Vs [Healthy]) :Normalized
1	Geraniol (106-24-1)	98	26	14.18871	1.65E-19	1.365947
2	Palmitic Acid (55520-89-3)	100	60	23.55566	0.002264404	0.6102581
3	α-Glyceryl Palmitate (1188-74-5)	100	49	27.26245	1.04E-06	0.8656348
4	Lactic Acid (17596-96-2)	70	26	9.07812	0.00308354	0.5949775
5	α-Glyceryl stearate (1188-75-6)	100	61	28.265228	7.23E-06	0.80729926
6	M-Pyrol (872-50-4)	53	1	8.527779	7.22E-11	1.082454
7	Citronellyl formate (105-85-1)	81	17	12.806128	4.35E-11	1.0938886
8	Sucrose (19159-25-2)	73	17	27.751137	3.00E-08	0.9526255
9	Triethanolamine (20836-42-4)	84	3	17.671268	1.88E-30	1.5848455
10	5-Ethyl-5-methyldecane (17312-74-2)	28	1	16.734486	3.86E-04	0.68131626
11	2,3-dimethyl-2,3-butane-diol (76-09-5)	57	6	11.471427	9.78E-09	0.97938067
12	Boric Acid (4325-85-3)	36	0	7.138889	1.26E-06	0.85958195
13	Phosphoric Acid (10497-05-9)	20	0	12.92	0.002640698	0.6035476
14	312.0@21.099983	72	26	21.099983	0.002248546	0.61144674
15	147.0@6.300002	39	0	6.300002	1.91E-07	0.9072522
16	151.0@6.0	32	3	6	6.99E-04	0.6602574
17	71.0@9.700005	45	5	9.700005	1.06E-05	0.7957854

**Table 2:** List of down-regulated metabolites in β-thalassemia patients in comparison to healthy controls.

S. No.	Compound (CAS No.)	Compound Found in No of Disease Samples/100	Compound Found in No of Healthy Samples/61	Retention Time	p (Corr) ([Disease] Vs [Healthy]) :Normalized	Log FC (abs) ([Disease] Vs [Healthy]) :Normalized
1	Hexadecane (544-76-3)	10	22	23.89688	9.40E-04	−0.6479106
2	Methylbis(phenylmethyl)benzene (999383-38-3)	0	14	25.5	6.50E-06	−0.8112275
3	Dodecane, 4,6-dimethyl (61141-72-8)	15	30	17.23111	3.91E-05	−0.75676084
4	Phthalic acid (117-81-7)	6	33	27.08717	1.49E-11	−1.11504
5	Glycerol (6787-10-6)	49	50	12.99899	0.001520142	−0.6268371
6	Stearic acid (57-11-4)	0	21	24.59524	1.61E-09	−1.0188797
7	n-Pentatriacontane (630-07-9)	3	14	20.94118	8.19E-04	−0.65371585
8	Ethylene glycol (7381-30-8)	0	14	7.350001	6.50E-06	−0.8115035
9	77.0@19.5	1	27	19.5	3.94E-12	−1.1401991
10	77.0@19.200005	0	22	19.20001	4.89E-10	−1.0456579
11	73.0@8.100001	1	21	8.100001	1.22E-08	−0.9731995
12	55.0@24.399998	0	9	24.4	0.001147105	−0.63986087
13	71.0@22.799995	5	22	22.8	3.09E-06	−0.8342981
14	71.0@25.899998	1	19	25.9	1.43E-07	−0.91528964
15	73.0@12.899999	2	11	12.9	0.002962446	−0.5973833
16	71.0@13.600004	11	25	13.6	1.45E-04	−0.7139679
17	57.0@24.600004	2	11	24.6	0.003284676	−0.59084994
18	69.0@12.799999	2	23	12.8	8.59E-09	−0.9838301
19	57.0@11.699998	1	17	11.7	2.23E-06	−0.8439079
20	73.0@14.199998	0	12	14.2	5.17E-05	−0.74642193
21	73.0@18.5:1	24	32	18.5	0.003341812	−0.5893601
22	73.0@9.199999	5	15	9.199999	0.003284676	−0.5910041
23	57.0@27.100002	0	8	27.1	0.00271492	−0.60159874

**Table 3:** Confusion Matrix of Model generated from healthy controls (n = 61) and β-thalassemia patients (n = 100).

	Β-Thalassemia Predicted	Healthy Predicted	Accuracy
True Β-Thalassemia	92	8	92.00
True Healthy	3	58	95.08
Overall Accuracy			93.17

**Table 4:** List of up-regulated and down-regulated pathways in β-thalassemia patients in comparison to healthy controls.

Pathway Name	Total Compound Present in Pathway	Hits	Raw p	−log(p)	Holm p	FDR	Impact
**Up-regulated:**							
Fatty acid elongation in mitochondria	27	1	0.0967	2.3362	1	1	0
Glycolysis or Gluconeogenesis	31	1	0.11029	2.2046	1	1	0
Pyruvate metabolism	32	1	0.11366	2.1745	1	1	0.13756
Propanoate metabolism	35	1	0.1237	2.0899	1	1	0
Glycerophospholipid metabolism	39	1	0.13694	1.9882	1	1	0
Galactose metabolism	41	1	0.14348	1.9415	1	1	0.01716
Fatty acid biosynthesis	49	1	0.16924	1.7764	1	1	0
Fatty acid metabolism	50	1	0.17241	1.7579	1	1	0.02959
Starch and sucrose metabolism	50	1	0.17241	1.7579	1	1	0.05947
**Down-regulated:**							
Glycerolipid metabolism	32	1	0.064781	2.7367	1	1	0.18847
Galactose metabolism	41	1	0.082382	2.4964	1	1	0
Fatty acid biosynthesis	49	1	0.097804	2.3248	1	1	0
Glyoxylate and dicarboxylate metabolism	50	1	0.099717	2.3054	1	1	0
