# Supplementary material for: β-Thalassemia Patients Revealed a Significant Change of Untargeted Metabolites in Comparison to Healthy Individuals
Source: Sci Rep. 2017 Feb 13;7:42249. doi: 10.1038/srep42249 (PMC5304209; doi:10.1038/srep42249)
Supplement: Supplementary Information [file srep42249-s1.pdf]

β-Thalassemia Patients Revealed a Significant Change of Untargeted Metabolites in Comparison to Healthy Individuals

Syed Ghulam Musharraf,[a,b] * Ayesha Iqbal,[b] Saqib Hussain Ansari,[c] Sadia Parveen,[c] Ishtiaq Ahmad Khan,[b] and Amna Jabbar Siddiqui[a]

[a] *H.E.J. Research Institute of Chemistry, International Center for Chemical and Biological Sciences, University of Karachi, Karachi-75270, Pakistan*

[b] *Dr. Panjwani Center for Molecular Medicine and Drug Research, International Center for Chemical and Biological Sciences, University of Karachi, Karachi-75270, Pakistan*

[c] *Department of Pediatric Hematology & Molecular Medicine, National Institute of Blood Diseases and Bone Marrow Transplantation, Karachi-75300, Pakistan*

* Corresponding author. Tel.: +92 21 34824924-5; 34819010; fax: + 92 21 34819018-9.
E-mail address: musharraf1977@yahoo.com

Table S1: Age, gender and weight of healthy normal subjects and β-thalassemia patients

S. No.	Parameter	β-Thalassemia Patients (n=100)	Healthy (n=61)
1.	Gender (Male/Female)	51/49	29/33
2.	Age (years)	9.13 ± 6.91	18.73 ± 7.93
3.	Weight (kgs)	22.41 ± 12.91	47.375 ± 16.34

Figure S1: The EI/MS spectra of unidentified compounds that are statistically differentially expressed between controls and β-thalassemia patients.

312.0@21.099983

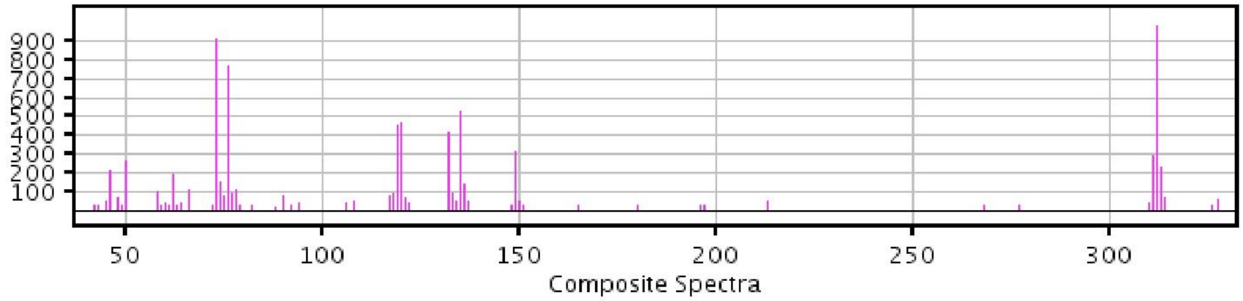

147.0@6.300002

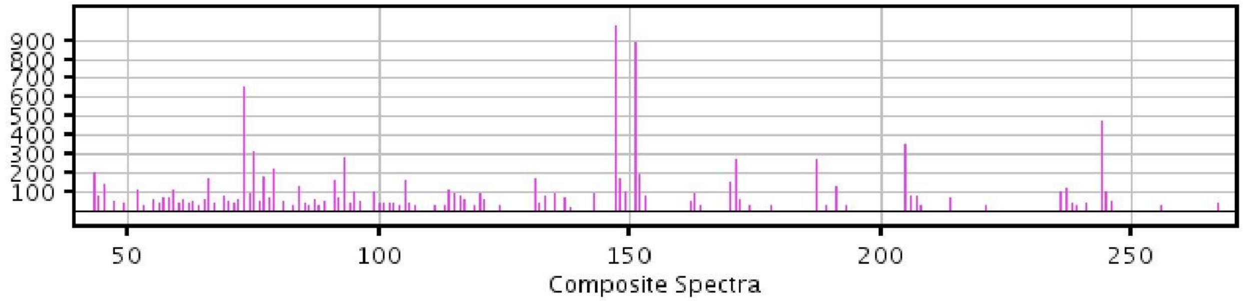

151.0@6.0

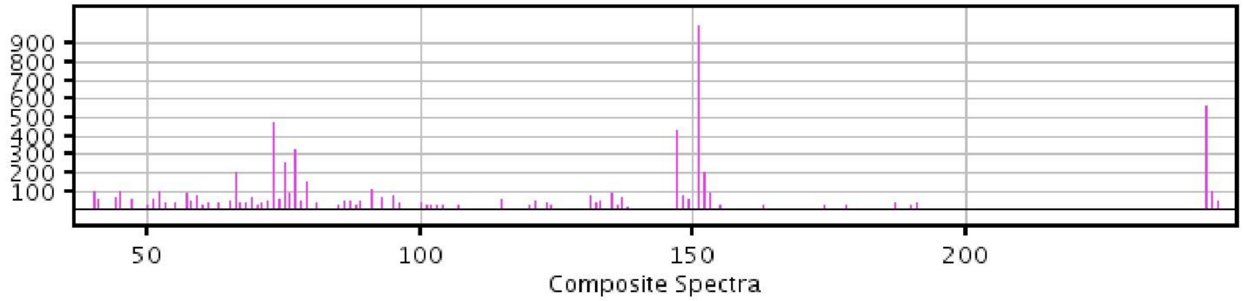

71.0@9.700005

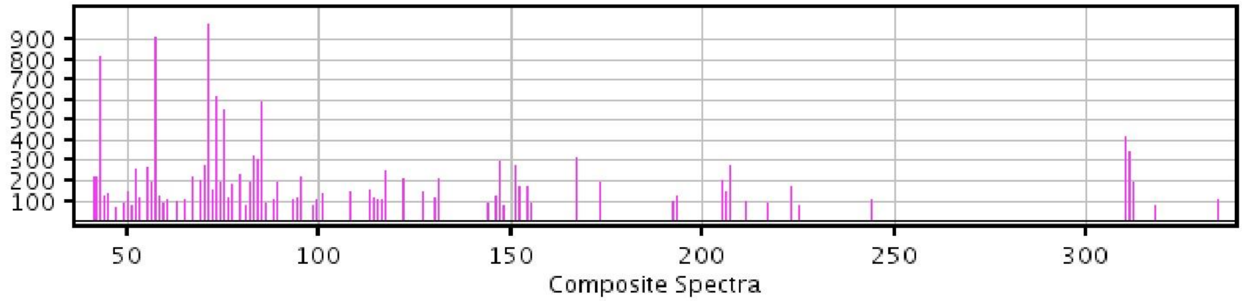

77.0@19.5

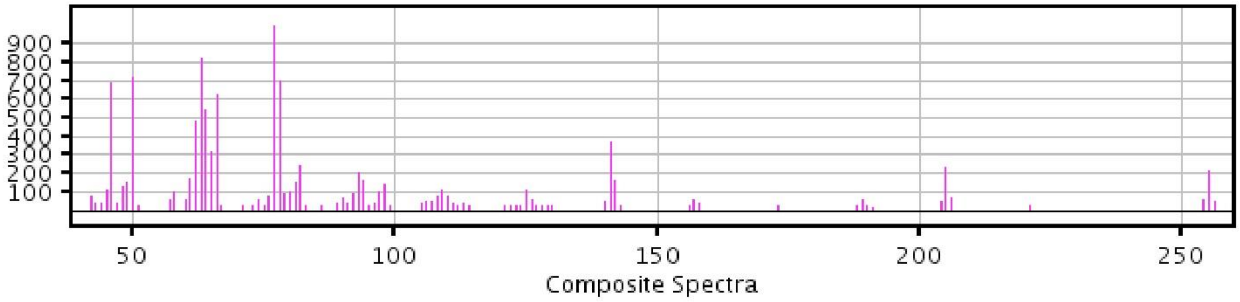

77.0@19.200005

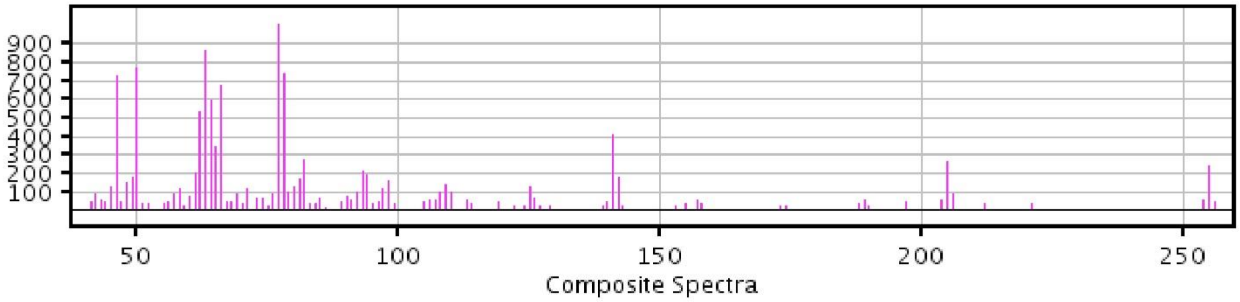

73.0@8.100001

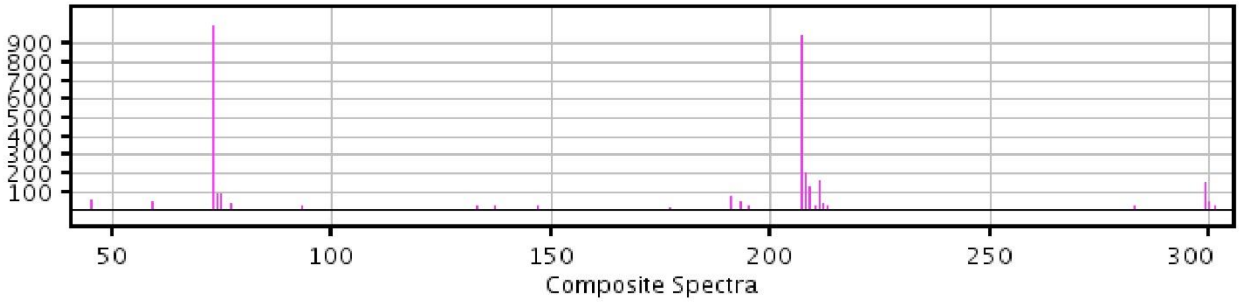

55.0@24.399998

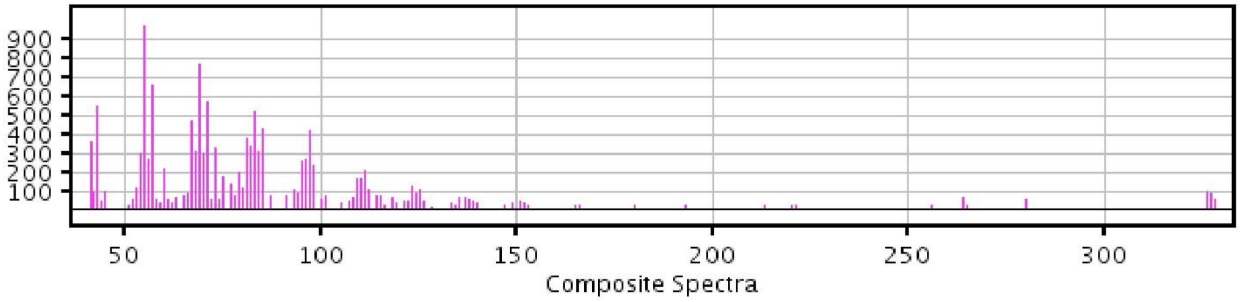

71.0@22.799995

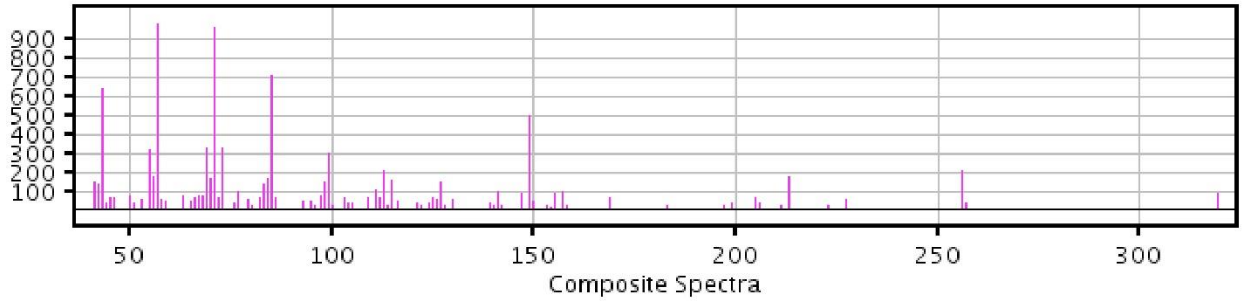

71.0@25.899998

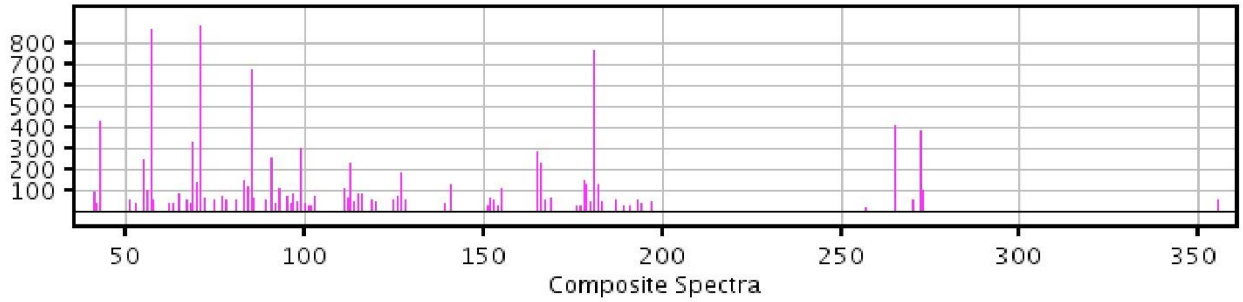

73.0@12.899999

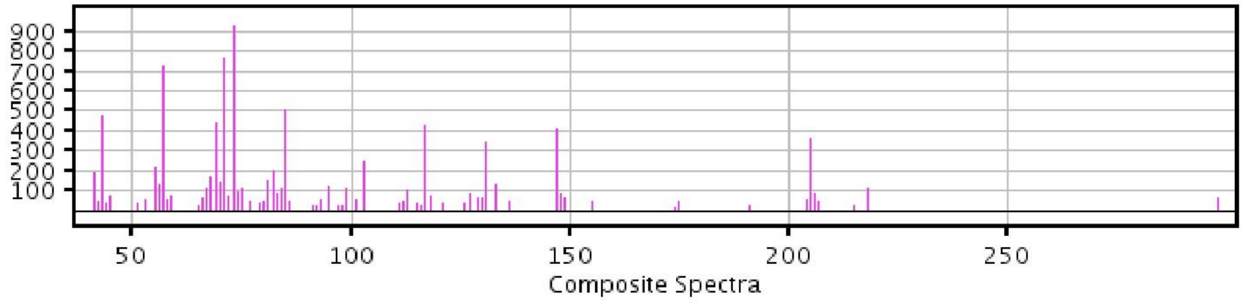

71.0@13.600004

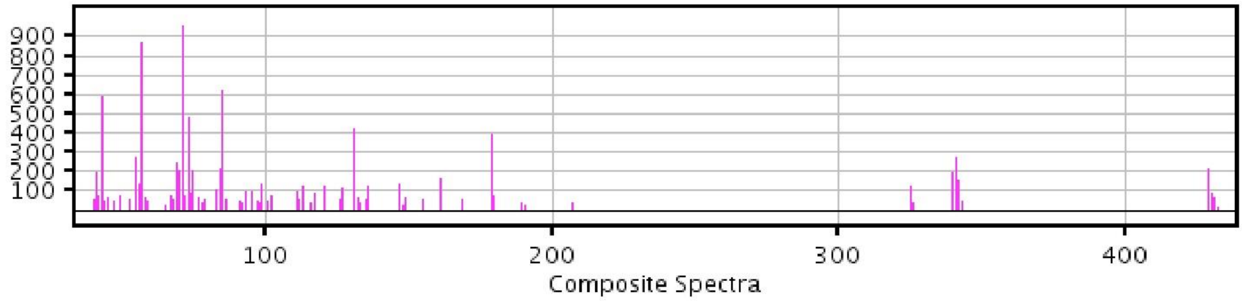

57.0@24.600004

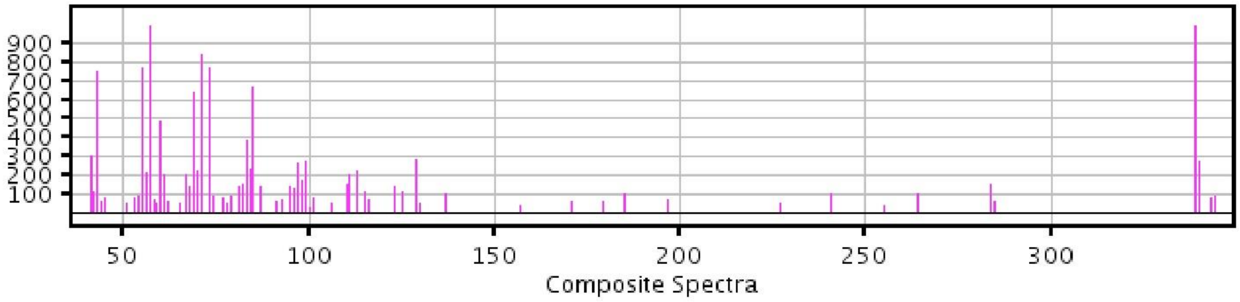

69.0@12.799999

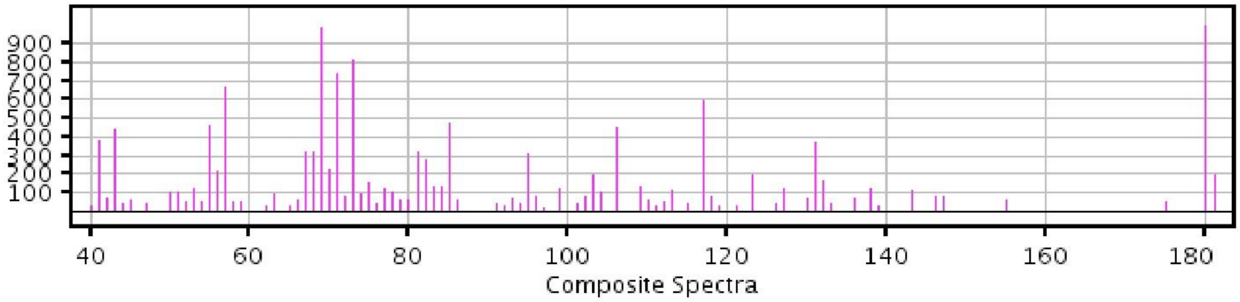

57.0@11.699998

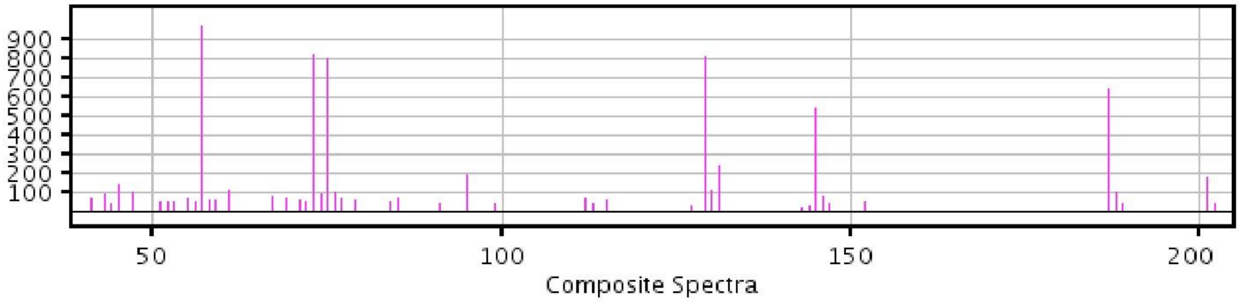

73.0@14.199998

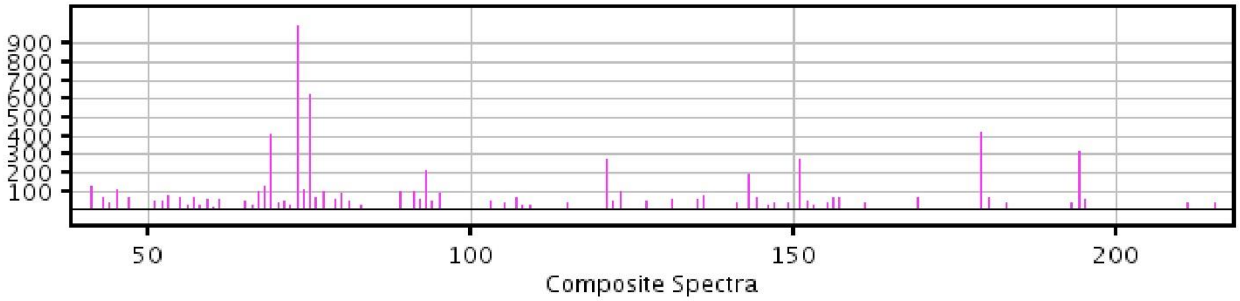

73.0@18.5:1

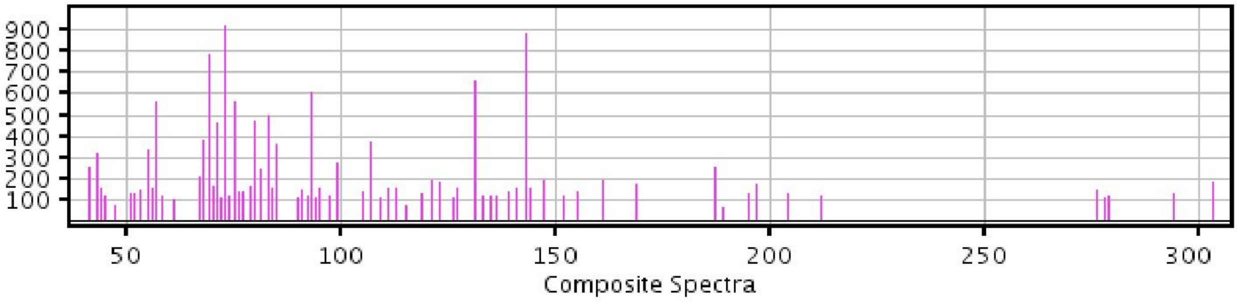

73.0@9.199999

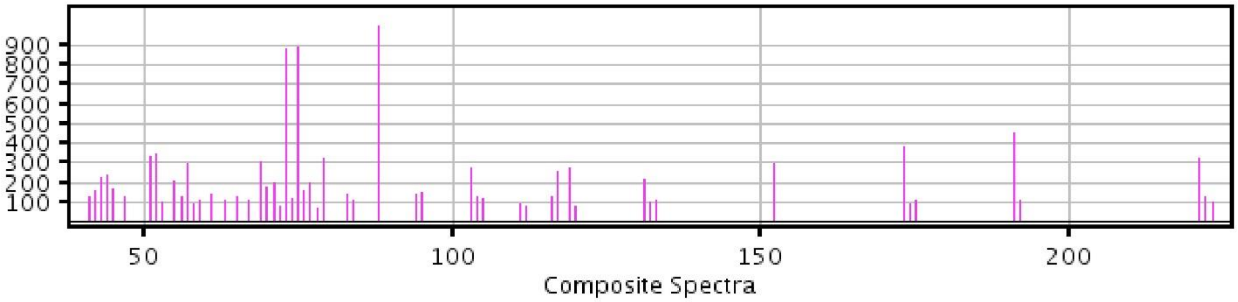

57.0@27.100002

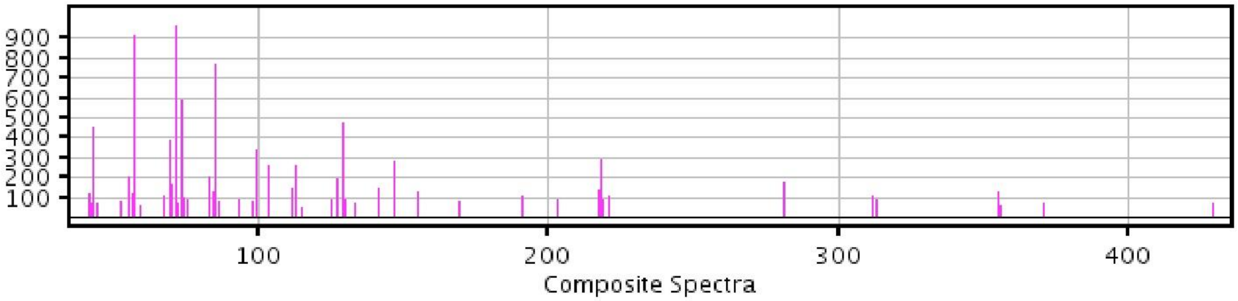

Figure S2: PCA plot showing that samples are not discriminating on the basis of age groups but due to health status (Group-0 = 0-20 years, Group-1 = 20-30 years). A) Down-regulated metabolites B) Up-regulated metabolites.

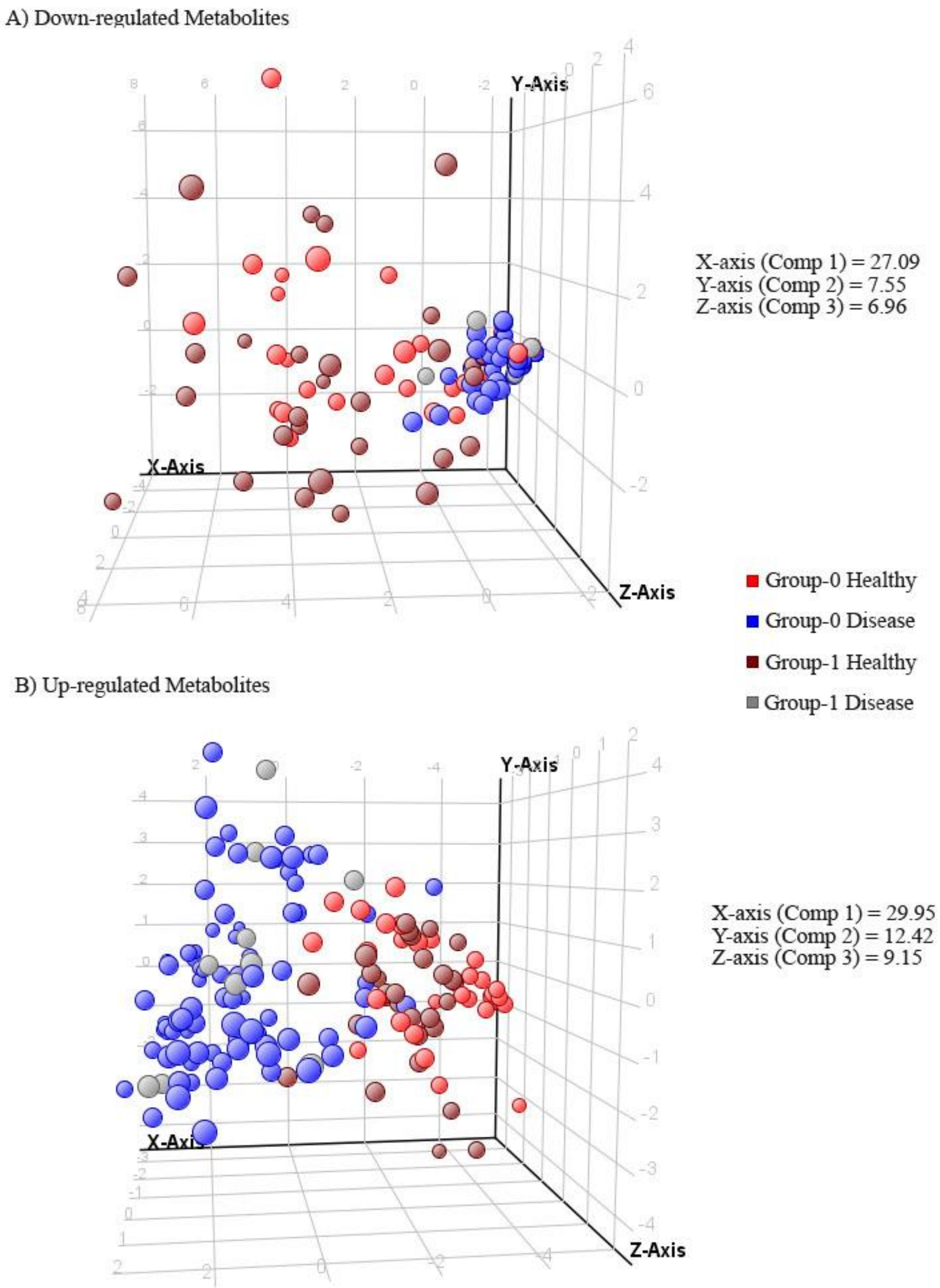

Figure S3: PCA plot showing that samples are not discriminating on the basis of body mass but due to health status. A) Down-regulated metabolites B) Up-regulated metabolites.

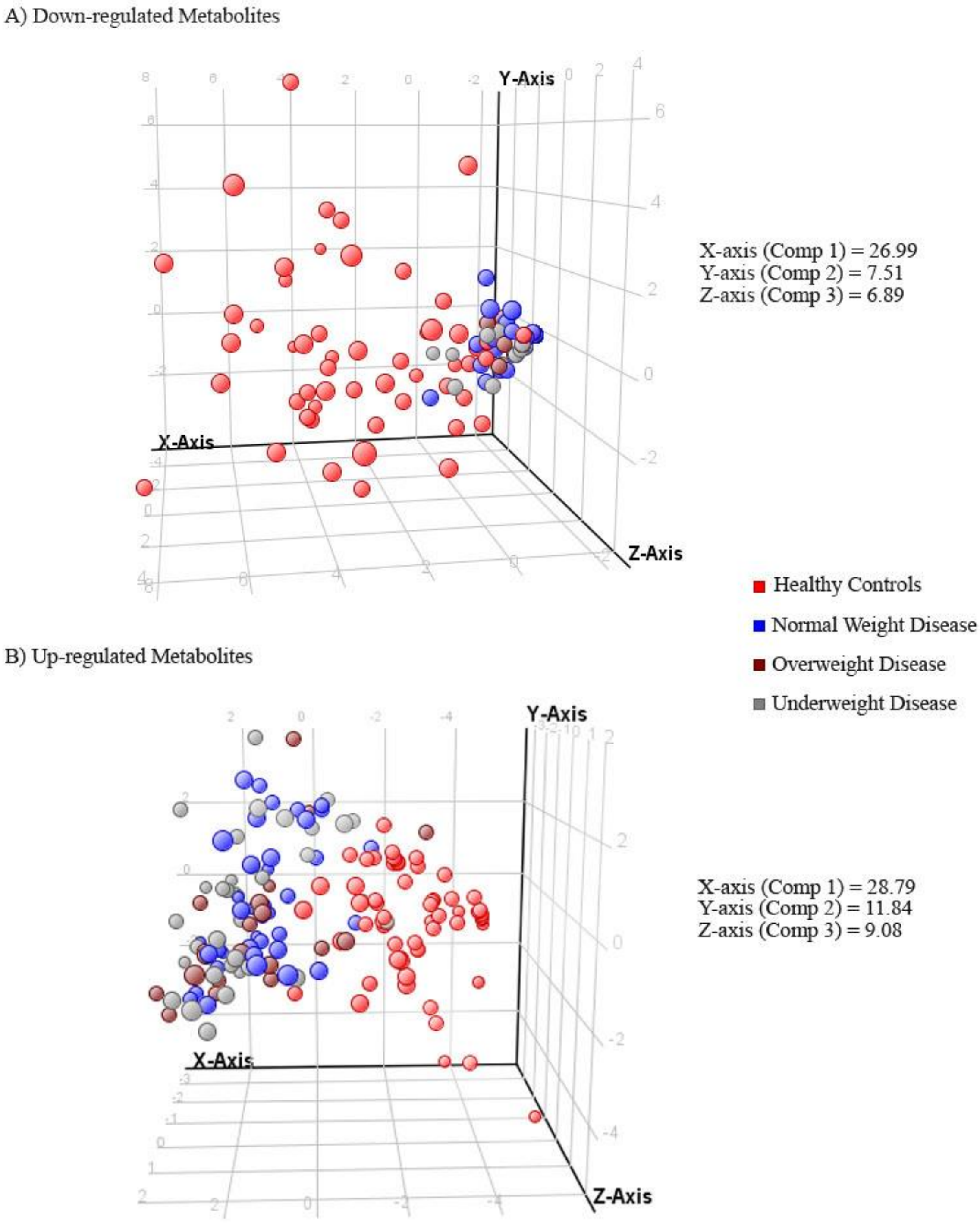

Figure S4: Model generated PCA scores scatter plots discriminating between healthy controls and β-thalassemia patients.

▲ Healthy Sample ■ Beta Thalassemia Sample

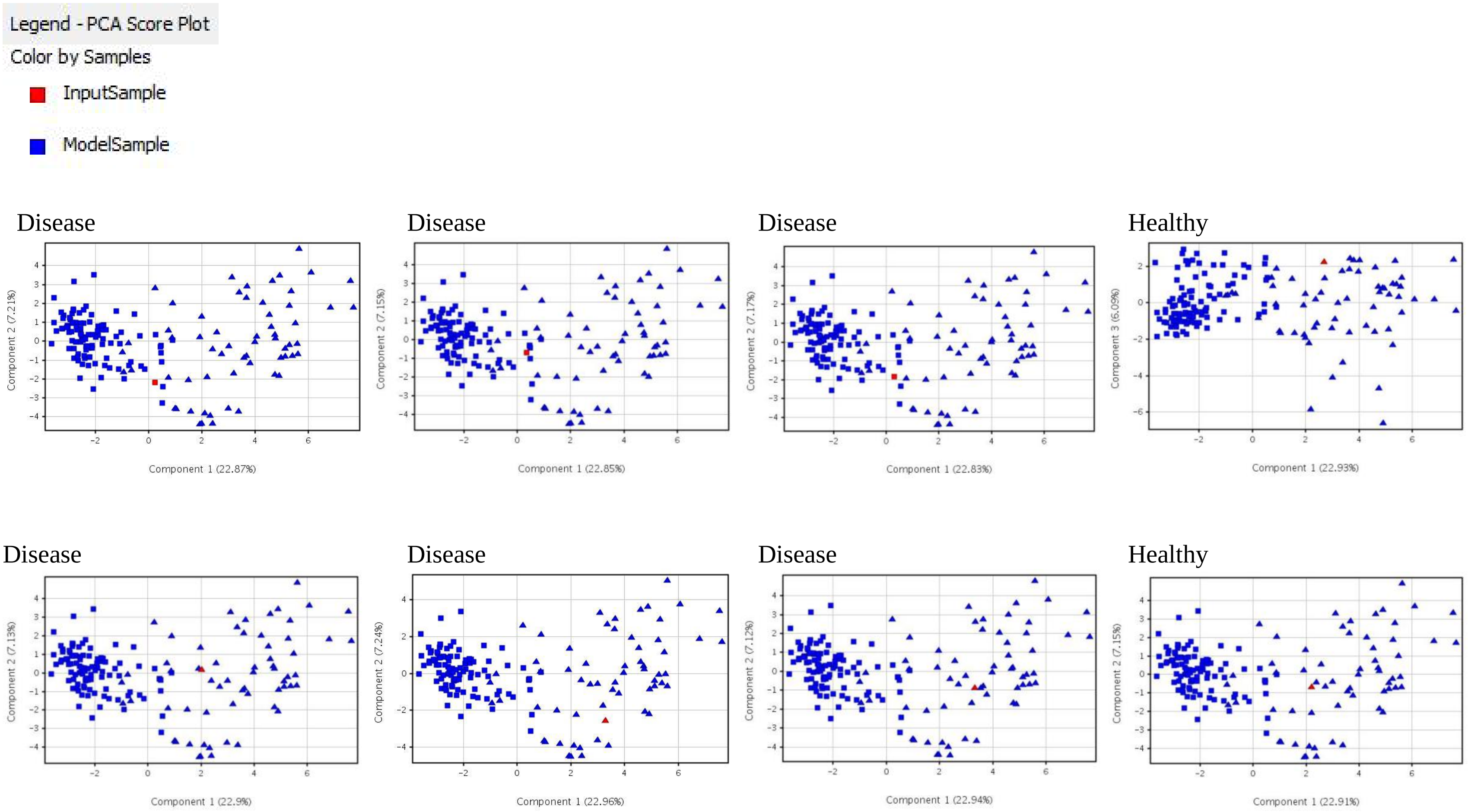

Disease

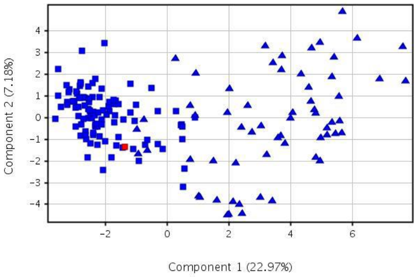

Healthy

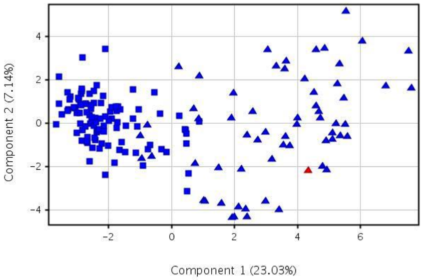

Healthy

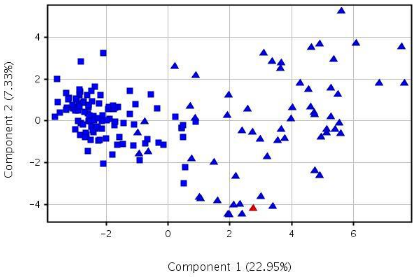

Healthy

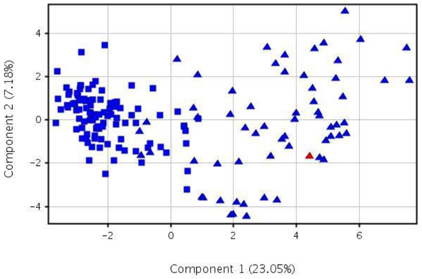

Healthy

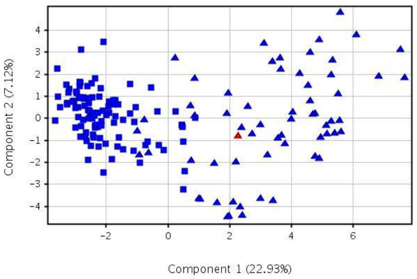

Disease

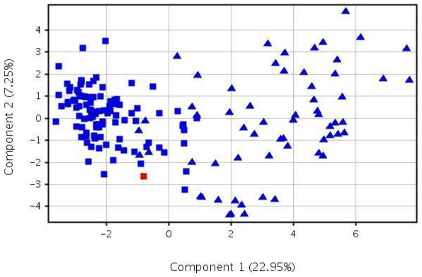

Disease

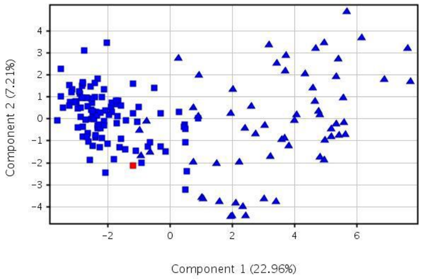

Disease

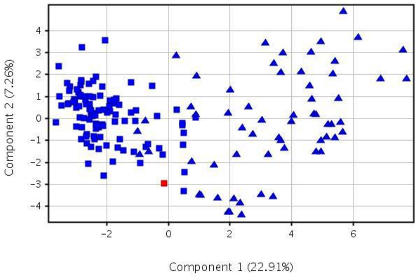

Healthy

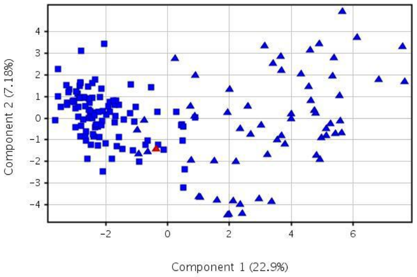

Healthy

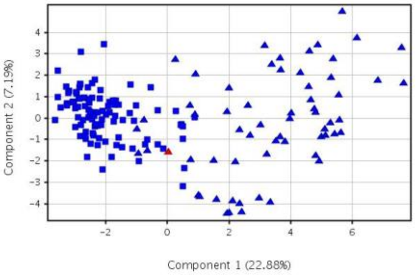

Healthy

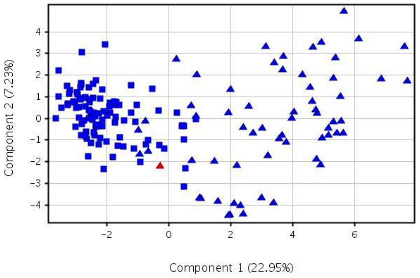

Healthy

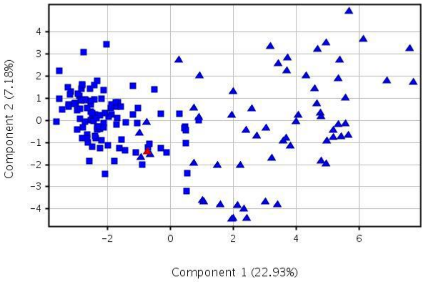

Figure S5: Summary of pathway analysis of metabolites that were up-regulated in β-thalassemia patients as compared to healthy controls.

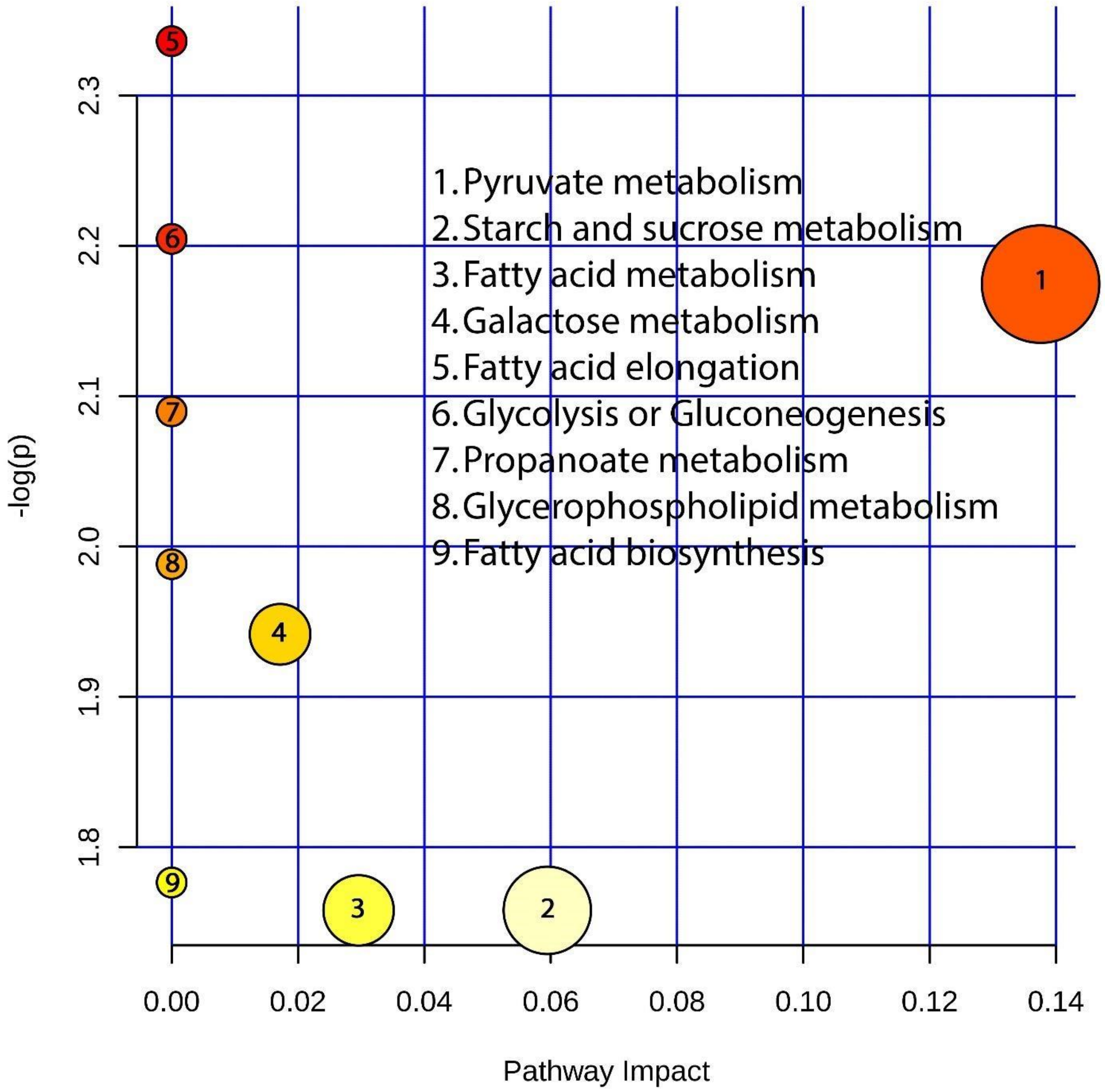

Figure S6: Summary of pathway analysis of metabolites that were down-regulated in β-thalassemia patients as compared to healthy controls

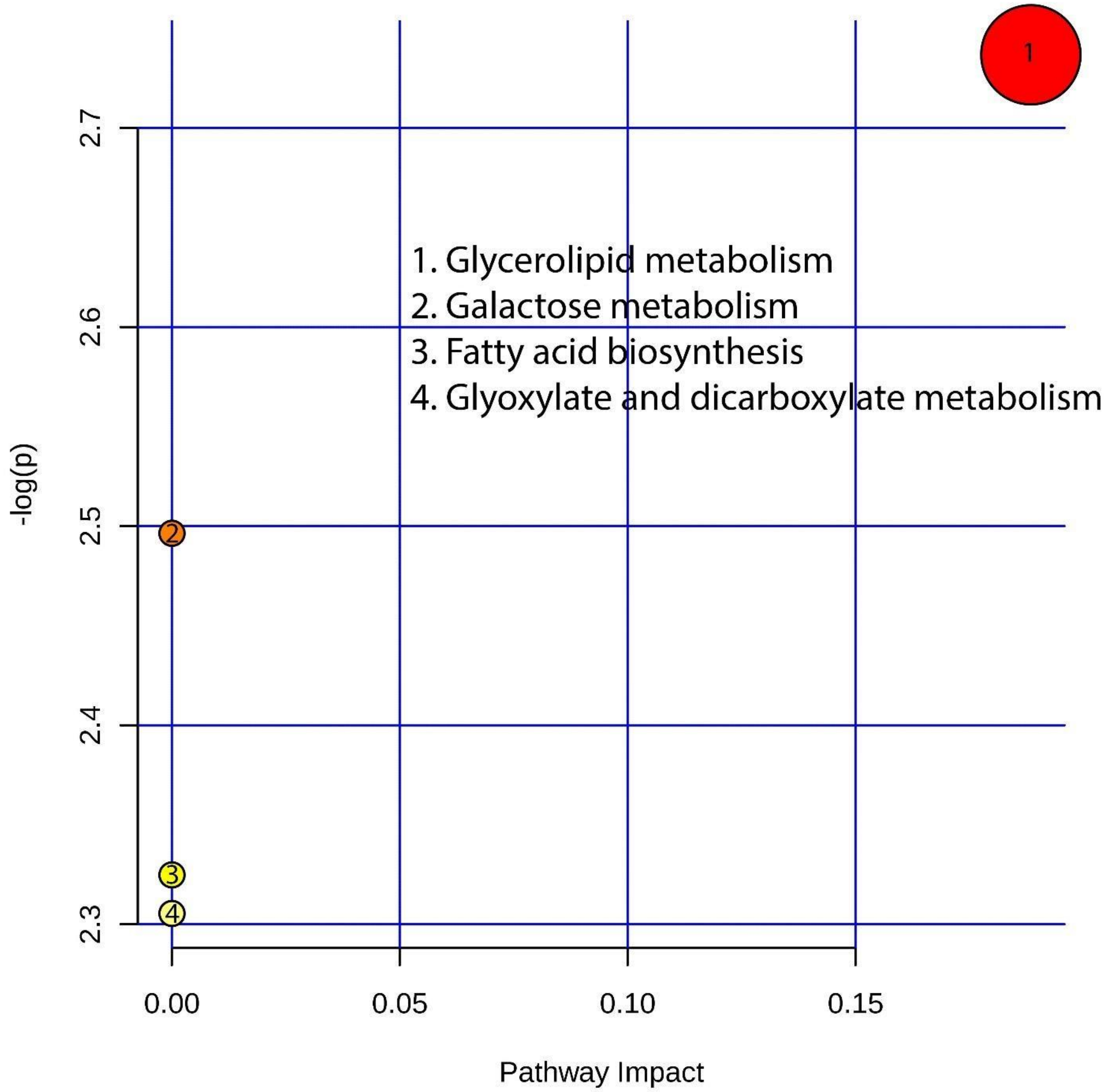

Figure S7: Pathways generated by list of metabolites discriminating between healthy controls and β-thalassemia patients.

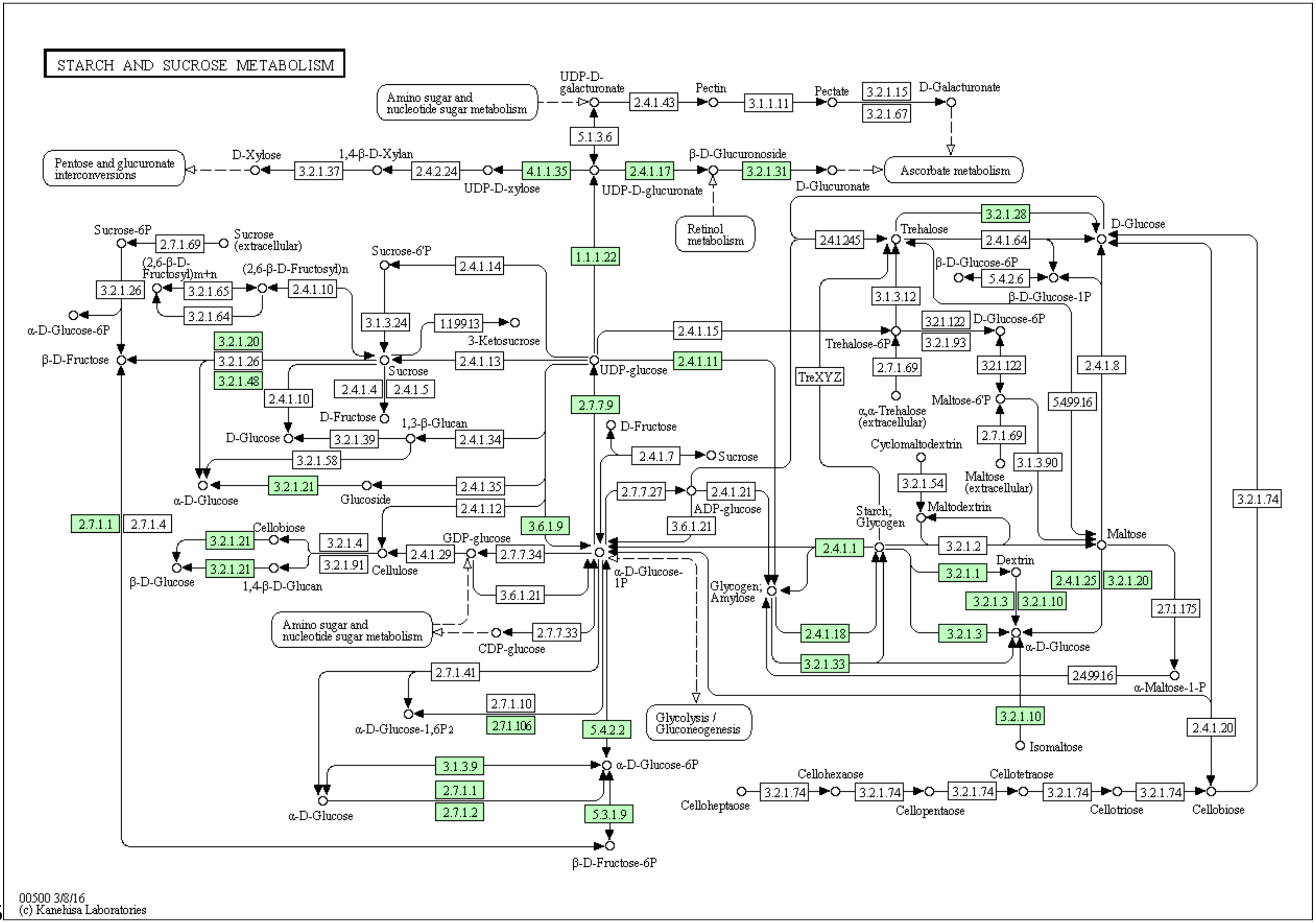

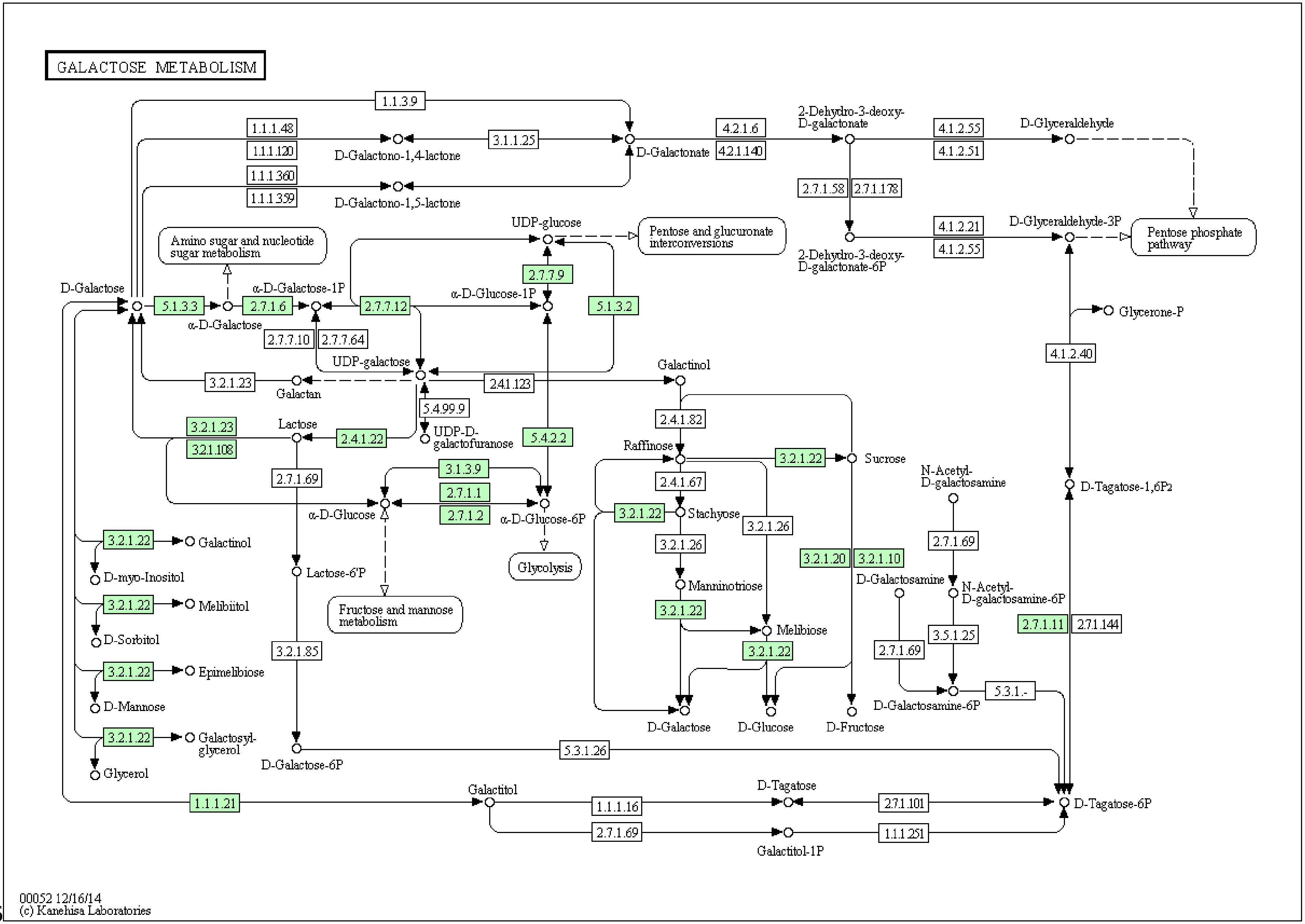
GALACTOSE METABOLISM
1.1.3.9
1.1.1.48
1.1.1.120
3.1.1.25
D-Galactono-1,4-lactone
D-Galactonate
4.2.1.6
4.2.1.140
2-Dehydro-3-deoxy-D-galactonate
4.1.2.55
4.1.2.51
D-Glyceraldehyde
1.1.1.360
1.1.1.359
D-Galactono-1,5-lactone
2.7.1.58
2.7.1.178
2-Dehydro-3-deoxy-D-galactonate-6P
4.1.2.21
4.1.2.55
D-Glyceraldehyde-3P
Pentose phosphate pathway
Amino sugar and nucleotide sugar metabolism
UDP-glucose
Pentose and glucuronate interconversions
2.7.7.9
D-Galactose
5.1.3.3
α-D-Galactose
2.7.1.6
α-D-Galactose-1P
2.7.7.12
α-D-Glucose-1P
5.1.3.2
Glycerone-P
2.7.7.10
2.7.7.64
4.1.2.40
UDP-galactose
3.2.1.23
Galactan
2.4.1.123
Galactinol
5.4.99.9
UDP-D-galactofuranose
3.2.1.23
3.2.1.108
Lactose
2.4.1.22
5.4.2.2
2.4.1.82
Raffinose
3.2.1.22
Sucrose
2.7.1.69
3.1.3.9
2.7.1.1
2.7.1.2
2.4.1.67
N-Acetyl-D-galactosamine
D-Tagatose-1,6P2
α-D-Glucose
α-D-Glucose-6P
3.2.1.22
Stachyose
3.2.1.26
Glycolysis
3.2.1.26
2.7.1.69
3.2.1.22
Galactinol
D-myo-Inositol
Lactose-6'P
3.2.1.20
3.2.1.10
D-Galactosamine
N-Acetyl-D-galactosamine-6P
Manninotriose
3.2.1.22
Melibiitol
D-Sorbitol
Fructose and mannose metabolism
3.2.1.22
Melibiose
2.7.1.11
2.7.1.144
3.5.1.25
2.7.1.69
3.2.1.85
3.2.1.22
3.2.1.22
Epimelibiose
D-Mannose
5.3.1.-
D-Galactose
D-Glucose
D-Fructose
D-Galactosamine-6P
3.2.1.22
Galactosyl-glycerol
Glycerol
D-Galactose-6P
5.3.1.26
1.1.1.21
Galactitol
1.1.1.16
D-Tagatose
2.7.1.101
D-Tagatose-6P
2.7.1.69
Galactitol-1P
1.1.1.251
00052 12/16/14
(c) Kanehisa Laboratories

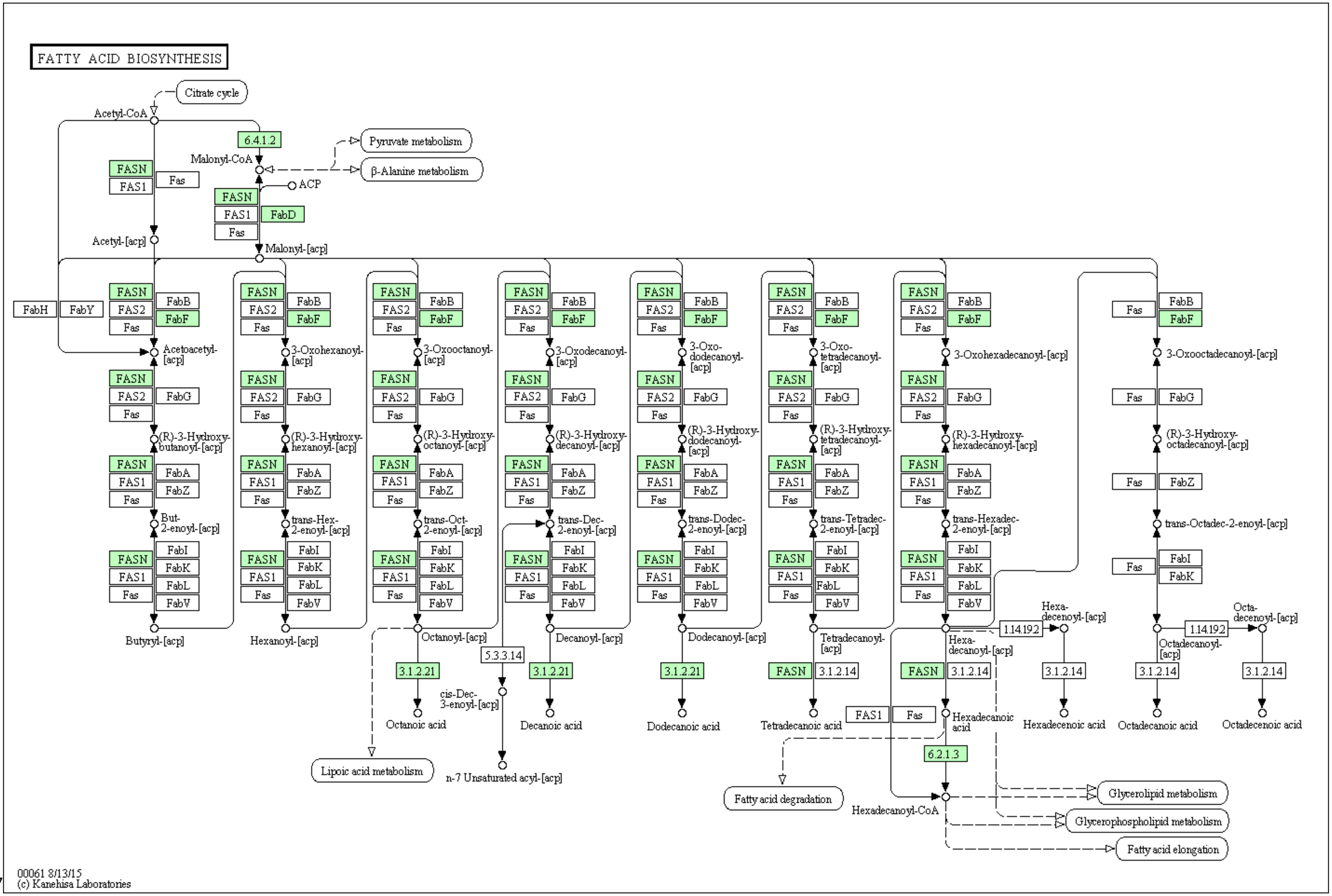
FATTY ACID BIOSYNTHESIS
Citrate cycle
Acetyl-CoA
6.4.1.2
Malonyl-CoA
Pyruvate metabolism
β-Alanine metabolism
ACP
FASN
FAS1
Fas
FabD
Acetyl-[acp]
Malonyl-[acp]
FabH
FabY
FAS2
FabB
FabF
FabG
FabA
FabZ
FabI
FabK
FabL
FabV
Acetoacetyl-[acp]
(R)-3-Hydroxy-butanoyl-[acp]
But-2-enoyl-[acp]
Butyryl-[acp]
3-Oxohexanoyl-[acp]
(R)-3-Hydroxy-hexanoyl-[acp]
trans-Hex-2-enoyl-[acp]
Hexanoyl-[acp]
3-Oxooctanoyl-[acp]
(R)-3-Hydroxy-octanoyl-[acp]
trans-Oct-2-enoyl-[acp]
Octanoyl-[acp]
3-Oxodecanoyl-[acp]
(R)-3-Hydroxy-decanoyl-[acp]
trans-Dec-2-enoyl-[acp]
Decanoyl-[acp]
3-Oxo-dodecanoyl-[acp]
(R)-3-Hydroxy-dodecanoyl-[acp]
trans-Dodec-2-enoyl-[acp]
Dodecanoyl-[acp]
3-Oxo-tetradecanoyl-[acp]
(R)-3-Hydroxy-tetradecanoyl-[acp]
trans-Tetradec-2-enoyl-[acp]
Tetradecanoyl-[acp]
3-Oxohexadecanoyl-[acp]
(R)-3-Hydroxy-hexadecanoyl-[acp]
trans-Hexadec-2-enoyl-[acp]
Hexa-decanoyl-[acp]
3-Oxooctadecanoyl-[acp]
(R)-3-Hydroxy-octadecanoyl-[acp]
trans-Octadec-2-enoyl-[acp]
Octadecanoyl-[acp]
Hexa-decenoyl-[acp]
Octa-decenoyl-[acp]
1.14.19.2
5.3.3.14
3.1.2.21
3.1.2.14
6.2.1.3
cis-Dec-3-enoyl-[acp]
n-7 Unsaturated acyl-[acp]
Octanoic acid
Decanoic acid
Dodecanoic acid
Tetradecanoic acid
Hexadecanoic acid
Hexadecenoic acid
Octadecanoic acid
Octadecenoic acid
Hexadecanoyl-CoA
Lipoic acid metabolism
Fatty acid degradation
Glycerolipid metabolism
Glycerophospholipid metabolism
Fatty acid elongation
00061 8/13/15
(c) Kanehisa Laboratories

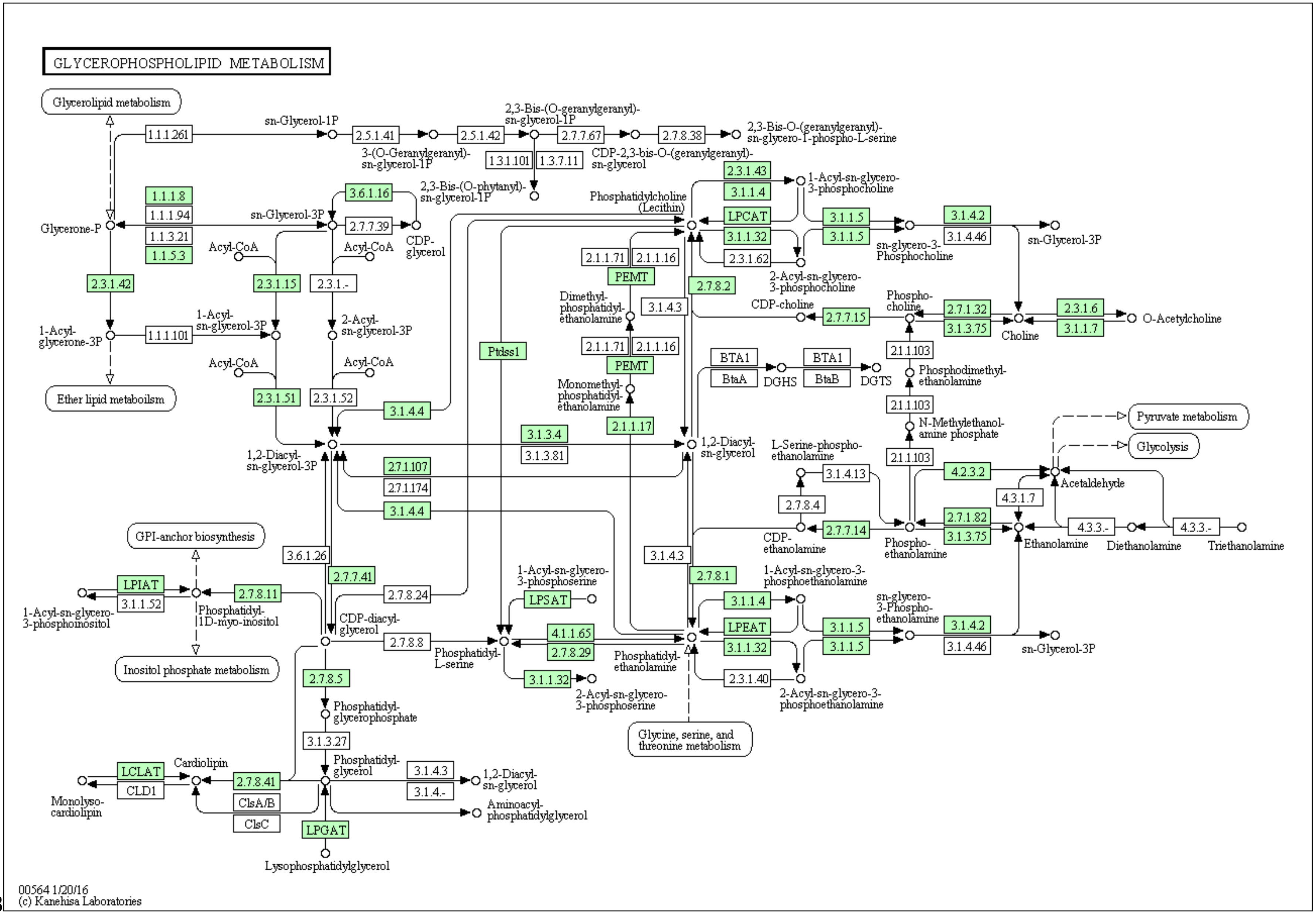
GLYCEROPHOSPHOLIPID METABOLISM
Glycerolipid metabolism
1.1.1.261
sn-Glycerol-1P
2.5.1.41
3-(O-Geranylgeranyl)-sn-glycerol-1P
2.5.1.42
2,3-Bis-(O-geranylgeranyl)-sn-glycerol-1P
1.3.1.101
1.3.7.11
2.7.7.67
CDP-2,3-bis-O-(geranylgeranyl)-sn-glycerol
2.7.8.38
2,3-Bis-O-(geranylgeranyl)-sn-glycero-1-phospho-L-serine
2,3-Bis-(O-phytanyl)-sn-glycerol-1P
1.1.1.8
1.1.1.94
1.1.3.21
1.1.5.3
Glycerone-P
sn-Glycerol-3P
3.6.1.16
2.7.7.39
CDP-glycerol
Acyl-CoA
2.3.1.42
2.3.1.15
2.3.1.-
1-Acyl-glycerone-3P
1.1.1.101
1-Acyl-sn-glycerol-3P
2-Acyl-sn-glycerol-3P
Ether lipid metaboilsm
2.3.1.51
2.3.1.52
3.1.4.4
1,2-Diacyl-sn-glycerol-3P
2.7.1.107
2.7.1.174
3.1.3.4
3.1.3.81
1,2-Diacyl-sn-glycerol
Ptdss1
Phosphatidylcholine (Lecithin)
2.3.1.43
3.1.1.4
LPCAT
3.1.1.32
2.3.1.62
1-Acyl-sn-glycero-3-phosphocholine
2-Acyl-sn-glycero-3-phosphocholine
3.1.1.5
sn-glycero-3-Phosphocholine
3.1.4.2
3.1.4.46
sn-Glycerol-3P
2.1.1.71
2.1.1.16
PEMT
Dimethyl-phosphatidyl-ethanolamine
3.1.4.3
2.7.8.2
CDP-choline
2.7.7.15
Phospho-choline
2.7.1.32
3.1.3.75
Choline
2.3.1.6
3.1.1.7
O-Acetylcholine
Monomethyl-phosphatidyl-ethanolamine
2.1.1.17
BTA1
BtaA
BtaB
DGHS
DGTS
2.1.1.103
Phosphodimethyl-ethanolamine
N-Methylethanol-amine phosphate
Pyruvate metabolism
Glycolysis
L-Serine-phospho-ethanolamine
3.1.4.13
4.2.3.2
4.3.1.7
Acetaldehyde
2.7.8.4
CDP-ethanolamine
2.7.7.14
2.7.1.82
Phospho-ethanolamine
Ethanolamine
4.3.3.-
Diethanolamine
Triethanolamine
GPI-anchor biosynthesis
3.6.1.26
2.7.7.41
2.7.8.24
3.1.4.3
2.7.8.1
LPIAT
3.1.1.52
2.7.8.11
1-Acyl-sn-glycero-3-phosphoinositol
Phosphatidyl-1D-myo-inositol
Inositol phosphate metabolism
CDP-diacyl-glycerol
2.7.8.8
1-Acyl-sn-glycero-3-phosphoserine
LPSAT
4.1.1.65
2.7.8.29
Phosphatidyl-L-serine
3.1.1.32
2-Acyl-sn-glycero-3-phosphoserine
Phosphatidyl-ethanolamine
LPEAT
2.3.1.40
1-Acyl-sn-glycero-3-phosphoethanolamine
2-Acyl-sn-glycero-3-phosphoethanolamine
sn-glycero-3-Phospho-ethanolamine
Glycine, serine, and threonine metabolism
2.7.8.5
Phosphatidyl-glycerophosphate
3.1.3.27
Phosphatidyl-glycerol
3.1.4.3
3.1.4.-
1,2-Diacyl-sn-glycerol
Aminoacyl-phosphatidylglycerol
LCLAT
CLD1
Cardiolipin
2.7.8.41
ClsA/B
ClsC
Monolyso-cardiolipin
LPGAT
Lysophosphatidylglycerol
00564 1/20/16
(c) Kanehisa Laboratories

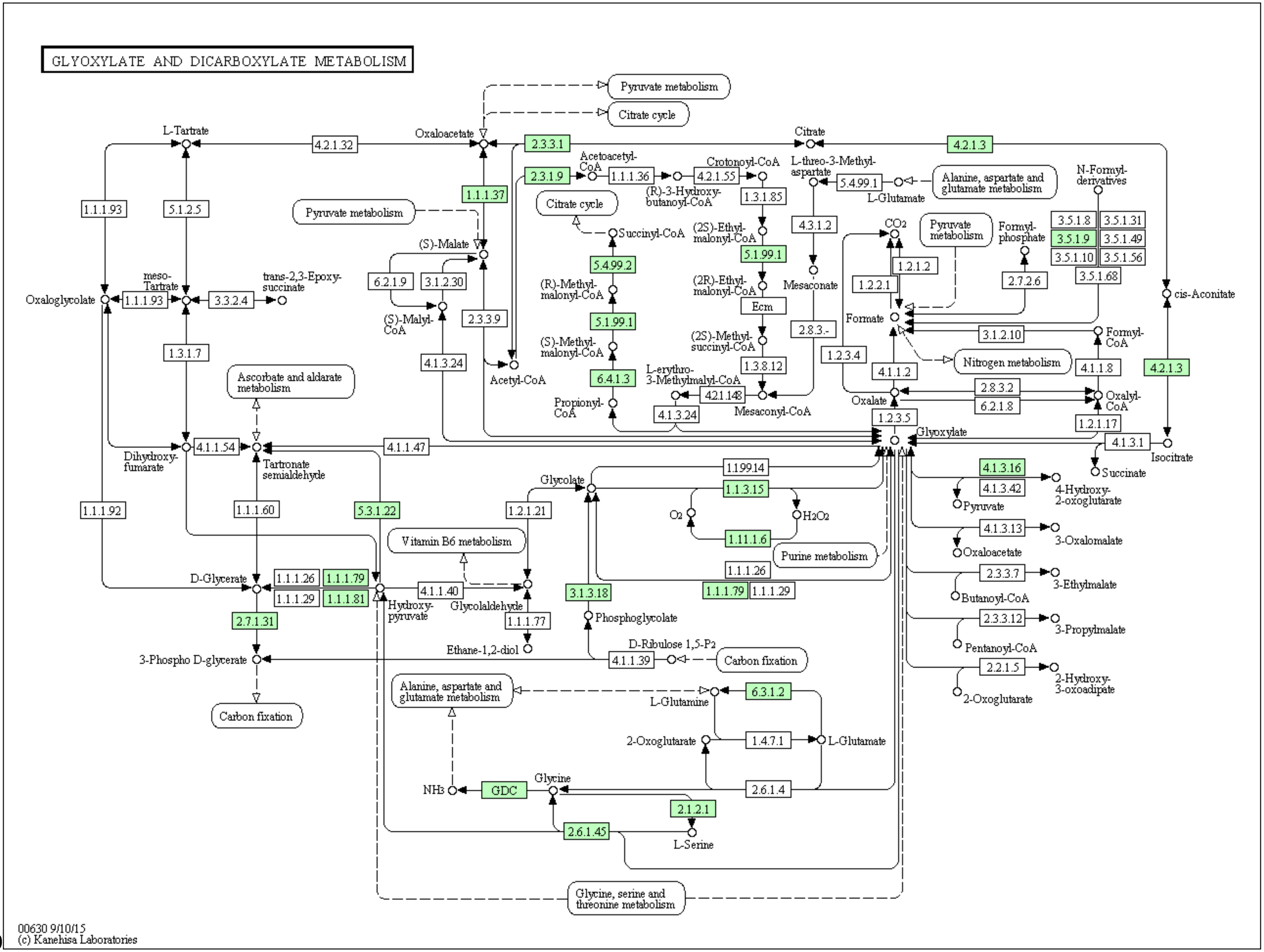
GLYOXYLATE AND DICARBOXYLATE METABOLISM
Pyruvate metabolism
Citrate cycle
L-Tartrate
4.2.1.32
Oxaloacetate
2.3.3.1
Citrate
4.2.1.3
Acetoacetyl-CoA
2.3.1.9
1.1.1.36
(R)-3-Hydroxy-butanoyl-CoA
4.2.1.55
Crotonoyl-CoA
L-threo-3-Methyl-aspartate
5.4.99.1
L-Glutamate
Alanine, aspartate and glutamate metabolism
N-Formyl-derivatives
1.1.1.93
5.1.2.5
1.1.1.37
Pyruvate metabolism
Citrate cycle
1.3.1.85
4.3.1.2
CO2
Pyruvate metabolism
Formyl-phosphate
3.5.1.8
3.5.1.31
3.5.1.9
3.5.1.49
3.5.1.10
3.5.1.56
3.5.1.68
(S)-Malate
Succinyl-CoA
(2S)-Ethyl-malonyl-CoA
5.1.99.1
1.2.1.2
meso-Tartrate
trans-2,3-Epoxy-succinate
6.2.1.9
3.1.2.30
5.4.99.2
(R)-Methyl-malonyl-CoA
(2R)-Ethyl-malonyl-CoA
Mesaconate
1.2.2.1
2.7.2.6
Oxaloglycolate
1.1.1.93
3.3.2.4
(S)-Malyl-CoA
2.3.3.9
5.1.99.1
Ecm
Formate
cis-Aconitate
(S)-Methyl-malonyl-CoA
(2S)-Methyl-succinyl-CoA
2.8.3.-
3.1.2.10
Formyl-CoA
1.3.1.7
4.1.3.24
Acetyl-CoA
6.4.1.3
1.3.8.12
1.2.3.4
Nitrogen metabolism
Ascorbate and aldarate metabolism
L-erythro-3-Methylmalyl-CoA
4.1.1.2
4.1.1.8
4.2.1.3
Propionyl-CoA
4.2.1.148
Mesaconyl-CoA
Oxalate
2.8.3.2
Oxalyl-CoA
4.1.3.24
6.2.1.8
1.2.3.5
1.2.1.17
Glyoxylate
4.1.1.54
4.1.1.47
4.1.3.1
Dihydroxy-fumarate
Tartronate semialdehyde
Isocitrate
Succinate
1.1.99.14
4.1.3.16
Glycolate
1.1.3.15
4-Hydroxy-2-oxoglutarate
4.1.3.42
Pyruvate
1.1.1.92
1.1.1.60
5.3.1.22
1.2.1.21
O2
H2O2
1.11.1.6
4.1.3.13
3-Oxalomalate
Oxaloacetate
Vitamin B6 metabolism
Purine metabolism
D-Glycerate
1.1.1.26
1.1.1.79
1.1.1.29
1.1.1.81
4.1.1.40
1.1.1.26
2.3.3.7
3-Ethylmalate
3.1.3.18
1.1.1.79
1.1.1.29
Butanoyl-CoA
Hydroxy-pyruvate
Glycolaldehyde
2.7.1.31
1.1.1.77
Phosphoglycolate
2.3.3.12
3-Propylmalate
Pentanoyl-CoA
Ethane-1,2-diol
D-Ribulose 1,5-P2
3-Phospho D-glycerate
4.1.1.39
Carbon fixation
2.2.1.5
2-Hydroxy-3-oxoadipate
Alanine, aspartate and glutamate metabolism
6.3.1.2
L-Glutamine
2-Oxoglutarate
Carbon fixation
2-Oxoglutarate
1.4.7.1
L-Glutamate
Glycine
NH3
GDC
2.6.1.4
2.1.2.1
2.6.1.45
L-Serine
Glycine, serine and threonine metabolism
00630 9/10/15
(c) Kanehisa Laboratories

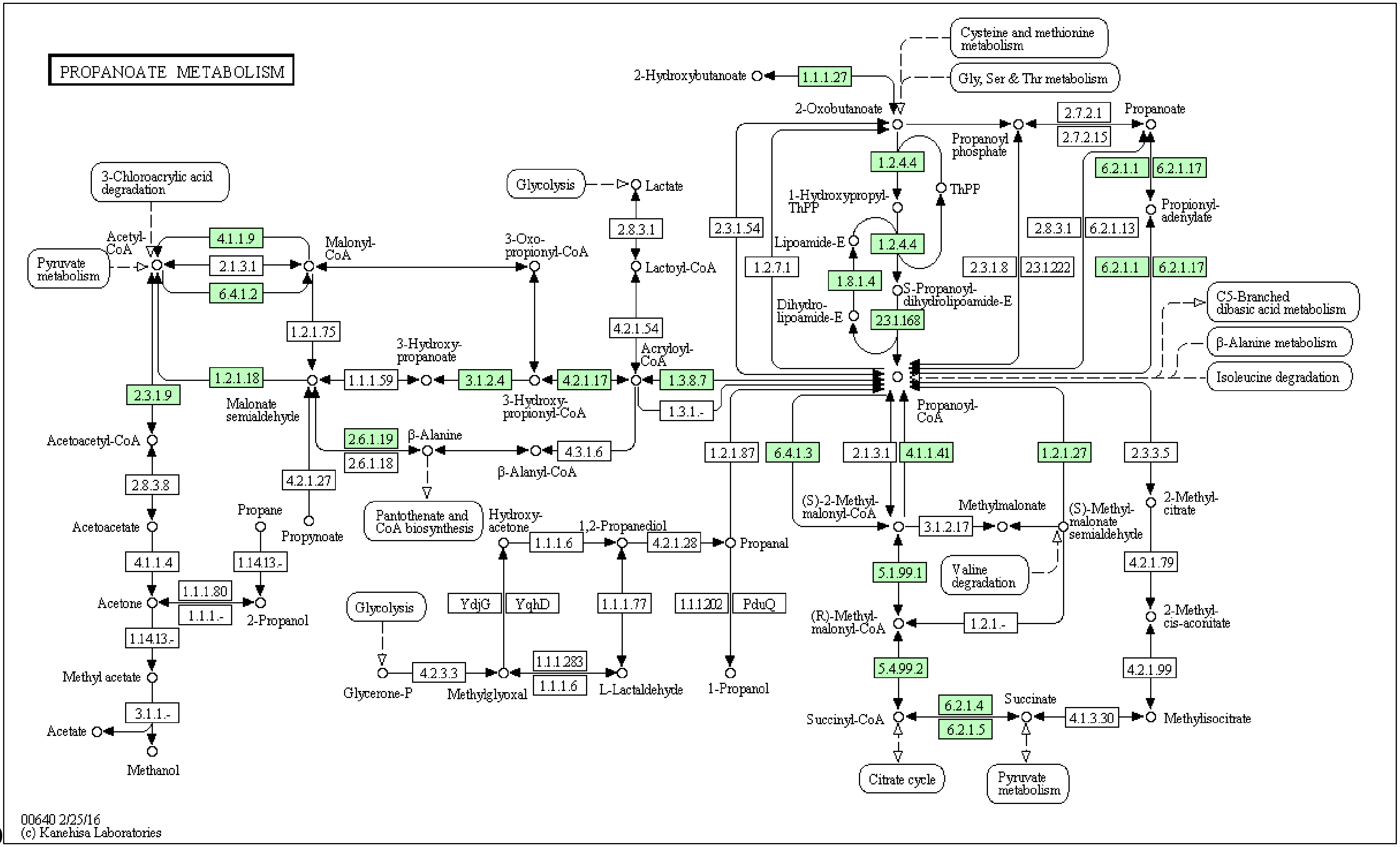

PROPANOATE METABOLISM
Cysteine and methionine metabolism
Gly, Ser & Thr metabolism
2-Hydroxybutanoate
1.1.1.27
2-Oxobutanoate
2.7.2.1
2.7.2.15
Propanoate
Propanoyl phosphate
1.2.4.4
ThPP
1-Hydroxypropyl-ThPP
6.2.1.1
6.2.1.17
Propionyl-adenylate
3-Chloroacrylic acid degradation
Glycolysis
Lactate
2.8.3.1
2.3.1.54
Lipoamide-E
1.2.4.4
2.8.3.1
6.2.1.13
Acetyl-CoA
4.1.1.9
Malonyl-CoA
3-Oxo-propionyl-CoA
Pyruvate metabolism
2.1.3.1
Lactoyl-CoA
1.2.7.1
1.8.1.4
2.3.1.8
2.3.1.222
6.2.1.1
6.2.1.17
6.4.1.2
S-Propanoyl-dihydrolipoamide-E
Dihydro-lipoamide-E
2.3.1.168
C5-Branched dibasic acid metabolism
1.2.1.75
4.2.1.54
3-Hydroxy-propanoate
Acryloyl-CoA
β-Alanine metabolism
1.2.1.18
1.1.1.59
3.1.2.4
4.2.1.17
1.3.8.7
Isoleucine degradation
2.3.1.9
Malonate semialdehyde
3-Hydroxy-propionyl-CoA
1.3.1.-
Propanoyl-CoA
2.6.1.19
β-Alanine
4.3.1.6
1.2.1.87
6.4.1.3
2.1.3.1
4.1.1.41
1.2.1.27
2.3.3.5
Acetoacetyl-CoA
2.6.1.18
β-Alanyl-CoA
2.8.3.8
4.2.1.27
Propane
Pantothenate and CoA biosynthesis
Hydroxy-acetone
1,2-Propanediol
(S)-2-Methyl-malonyl-CoA
Methylmalonate
(S)-Methyl-malonate semialdehyde
2-Methyl-citrate
Acetoacetate
Propynoate
1.1.1.6
4.2.1.28
Propanal
3.1.2.17
4.1.1.4
1.14.13.-
Valine degradation
5.1.99.1
4.2.1.79
1.1.1.80
Acetone
2-Propanol
1.1.1.-
Glycolysis
YdjG
YqhD
1.1.1.77
1.1.1.202
PduQ
(R)-Methyl-malonyl-CoA
1.2.1.-
2-Methyl-cis-aconitate
1.14.13.-
4.2.3.3
1.1.1.283
1.1.1.6
5.4.99.2
4.2.1.99
Methyl acetate
Glycerone-P
Methylglyoxal
L-Lactaldehyde
1-Propanol
Succinyl-CoA
6.2.1.4
6.2.1.5
Succinate
4.1.3.30
Methylisocitrate
3.1.1.-
Acetate
Methanol
Citrate cycle
Pyruvate metabolism
00640 2/25/16
(c) Kanehisa Laboratories

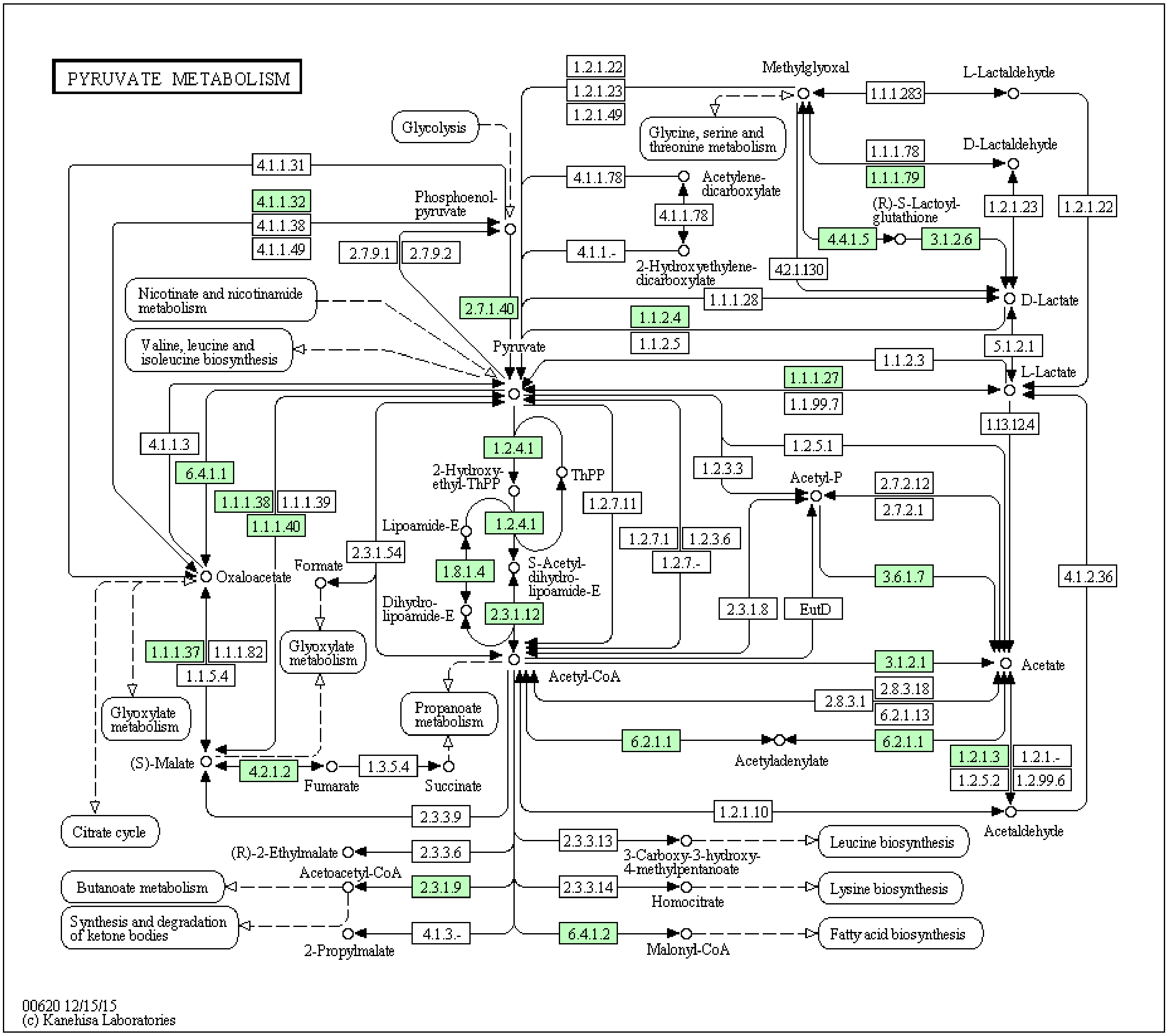
PYRUVATE METABOLISM
Glycolysis
Phosphoenol-pyruvate
Pyruvate
Oxaloacetate
Formate
(S)-Malate
Fumarate
Succinate
Acetyl-CoA
2-Hydroxy-ethyl-ThPP
ThPP
Lipoamide-E
S-Acetyl-dihydro-lipoamide-E
Dihydro-lipoamide-E
Methylglyoxal
L-Lactaldehyde
D-Lactaldehyde
(R)-S-Lactoyl-glutathione
D-Lactate
L-Lactate
Acetylene-dicarboxylate
2-Hydroxyethylene-dicarboxylate
Acetyl-P
Acetate
Acetyladenylate
Acetaldehyde
(R)-2-Ethylmalate
Acetoacetyl-CoA
2-Propylmalate
3-Carboxy-3-hydroxy-4-methylpentanoate
Homocitrate
Malonyl-CoA
Glycine, serine and threonine metabolism
Nicotinate and nicotinamide metabolism
Valine, leucine and isoleucine biosynthesis
Glyoxylate metabolism
Glyoxylate metabolism
Propanoate metabolism
Citrate cycle
Butanoate metabolism
Synthesis and degradation of ketone bodies
Leucine biosynthesis
Lysine biosynthesis
Fatty acid biosynthesis
1.2.1.22
1.2.1.23
1.2.1.49
1.1.1.283
1.1.1.78
1.1.1.79
1.2.1.23
1.2.1.22
4.1.1.31
4.1.1.32
4.1.1.38
4.1.1.49
4.1.1.78
4.1.1.78
4.1.1.-
2.7.9.1
2.7.9.2
4.4.1.5
3.1.2.6
4.2.1.130
2.7.1.40
1.1.1.28
1.1.2.4
1.1.2.5
5.1.2.1
1.1.2.3
1.1.1.27
1.1.99.7
1.13.12.4
4.1.1.3
6.4.1.1
1.1.1.38
1.1.1.39
1.1.1.40
1.2.4.1
1.2.4.1
1.8.1.4
2.3.1.12
2.3.1.54
1.2.7.11
1.2.7.1
1.2.3.6
1.2.7.-
1.2.3.3
1.2.5.1
2.7.2.12
2.7.2.1
3.6.1.7
2.3.1.8
EutD
4.1.2.36
1.1.1.37
1.1.1.82
1.1.5.4
4.2.1.2
1.3.5.4
3.1.2.1
2.8.3.18
2.8.3.1
6.2.1.13
6.2.1.1
6.2.1.1
1.2.1.3
1.2.1.-
1.2.5.2
1.2.99.6
1.2.1.10
2.3.3.9
2.3.3.6
2.3.1.9
4.1.3.-
2.3.3.13
2.3.3.14
6.4.1.2
00620 12/15/15
(c) Kanehisa Laboratories

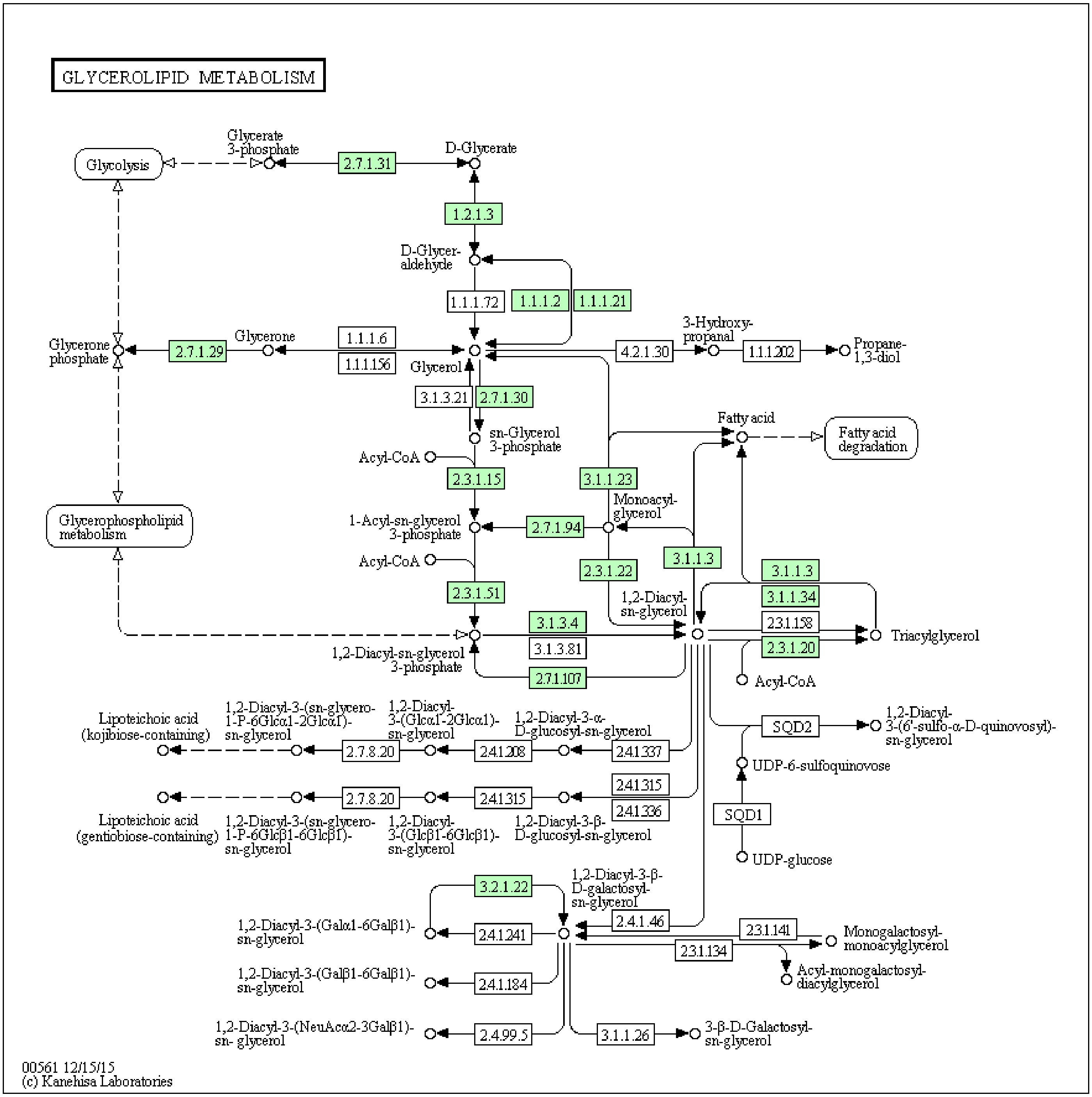
GLYCEROLIPID METABOLISM
Glycolysis
Glycerate 3-phosphate
2.7.1.31
D-Glycerate
1.2.1.3
D-Glycer-aldehyde
1.1.1.72
1.1.1.2
1.1.1.21
Glycerone phosphate
2.7.1.29
Glycerone
1.1.1.6
1.1.1.156
Glycerol
4.2.1.30
3-Hydroxy-propanal
1.1.1.202
Propane-1,3-diol
3.1.3.21
2.7.1.30
sn-Glycerol 3-phosphate
Fatty acid
Fatty acid degradation
Acyl-CoA
2.3.1.15
3.1.1.23
Glycerophospholipid metabolism
1-Acyl-sn-glycerol 3-phosphate
2.7.1.94
Monoacyl-glycerol
Acyl-CoA
3.1.1.3
3.1.1.3
2.3.1.22
2.3.1.51
3.1.1.34
1,2-Diacyl-sn-glycerol
3.1.3.4
2.3.1.158
Triacylglycerol
3.1.3.81
2.3.1.20
1,2-Diacyl-sn-glycerol 3-phosphate
2.7.1.107
Acyl-CoA
Lipoteichoic acid (kojibiose-containing)
1,2-Diacyl-3-(sn-glycero-1-P-6Glcα1-2Glcα1)-sn-glycerol
1,2-Diacyl-3-(Glcα1-2Glcα1)-sn-glycerol
1,2-Diacyl-3-α-D-glucosyl-sn-glycerol
SQD2
1,2-Diacyl-3-(6'-sulfo-α-D-quinovosyl)-sn-glycerol
2.7.8.20
2.4.1.208
2.4.1.337
UDP-6-sulfoquinovose
2.4.1.315
2.7.8.20
2.4.1.315
2.4.1.336
SQD1
Lipoteichoic acid (gentiobiose-containing)
1,2-Diacyl-3-(sn-glycero-1-P-6Glcβ1-6Glcβ1)-sn-glycerol
1,2-Diacyl-3-(Glcβ1-6Glcβ1)-sn-glycerol
1,2-Diacyl-3-β-D-glucosyl-sn-glycerol
UDP-glucose
3.2.1.22
1,2-Diacyl-3-β-D-galactosyl-sn-glycerol
1,2-Diacyl-3-(Galα1-6Galβ1)-sn-glycerol
2.4.1.241
2.4.1.46
2.3.1.141
Monogalactosyl-monoacylglycerol
2.3.1.134
Acyl-monogalactosyl-diacylglycerol
1,2-Diacyl-3-(Galβ1-6Galβ1)-sn-glycerol
2.4.1.184
1,2-Diacyl-3-(NeuAcα2-3Galβ1)-sn- glycerol
2.4.99.5
3.1.1.26
3-β-D-Galactosyl-sn-glycerol
00561 12/15/15
(c) Kanehisa Laboratories

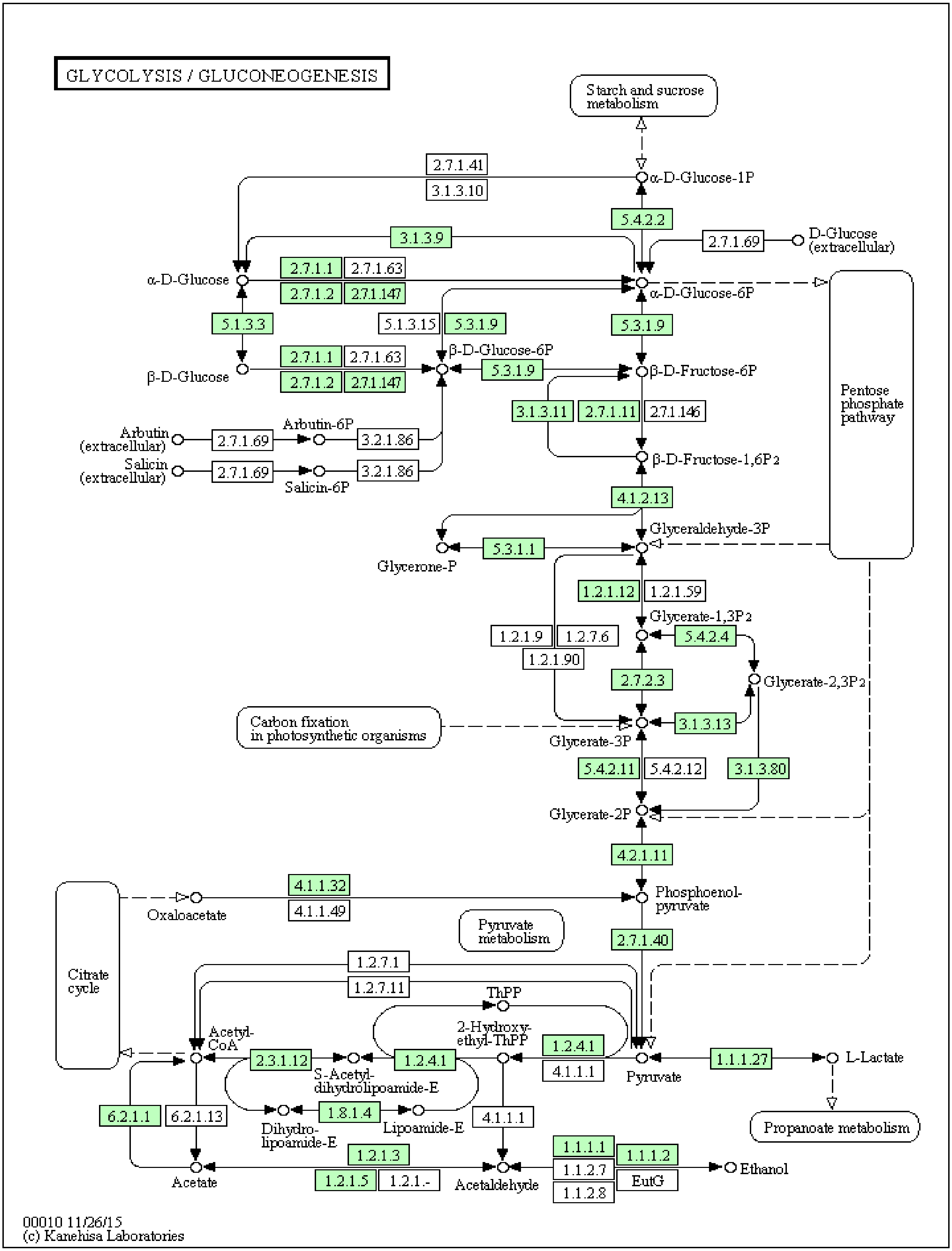
GLYCOLYSIS / GLUCONEOGENESIS
Starch and sucrose metabolism
2.7.1.41
3.1.3.10
α-D-Glucose-1P
5.4.2.2
3.1.3.9
2.7.1.69
D-Glucose (extracellular)
α-D-Glucose
2.7.1.1
2.7.1.63
2.7.1.2
2.7.1.147
α-D-Glucose-6P
5.1.3.3
5.1.3.15
5.3.1.9
5.3.1.9
β-D-Glucose-6P
β-D-Glucose
2.7.1.1
2.7.1.63
2.7.1.2
2.7.1.147
5.3.1.9
β-D-Fructose-6P
Pentose phosphate pathway
3.1.3.11
2.7.1.11
2.7.1.146
Arbutin (extracellular)
2.7.1.69
Arbutin-6P
3.2.1.86
Salicin (extracellular)
2.7.1.69
Salicin-6P
3.2.1.86
β-D-Fructose-1,6P2
4.1.2.13
Glyceraldehyde-3P
5.3.1.1
Glycerone-P
1.2.1.12
1.2.1.59
Glycerate-1,3P2
1.2.1.9
1.2.7.6
1.2.1.90
5.4.2.4
2.7.2.3
Glycerate-2,3P2
Carbon fixation in photosynthetic organisms
3.1.3.13
Glycerate-3P
5.4.2.11
5.4.2.12
3.1.3.80
Glycerate-2P
4.2.1.11
Citrate cycle
4.1.1.32
4.1.1.49
Oxaloacetate
Phosphoenol-pyruvate
Pyruvate metabolism
2.7.1.40
1.2.7.1
1.2.7.11
ThPP
2-Hydroxy-ethyl-ThPP
Acetyl-CoA
2.3.1.12
1.2.4.1
1.2.4.1
4.1.1.1
Pyruvate
1.1.1.27
L-Lactate
S-Acetyl-dihydrolipoamide-E
Dihydro-lipoamide-E
1.8.1.4
Lipoamide-E
4.1.1.1
Propanoate metabolism
6.2.1.1
6.2.1.13
1.2.1.3
1.2.1.5
1.2.1.-
1.1.1.1
1.1.2.7
1.1.2.8
1.1.1.2
EutG
Ethanol
Acetate
Acetaldehyde
00010 11/26/15
(c) Kanehisa Laboratories

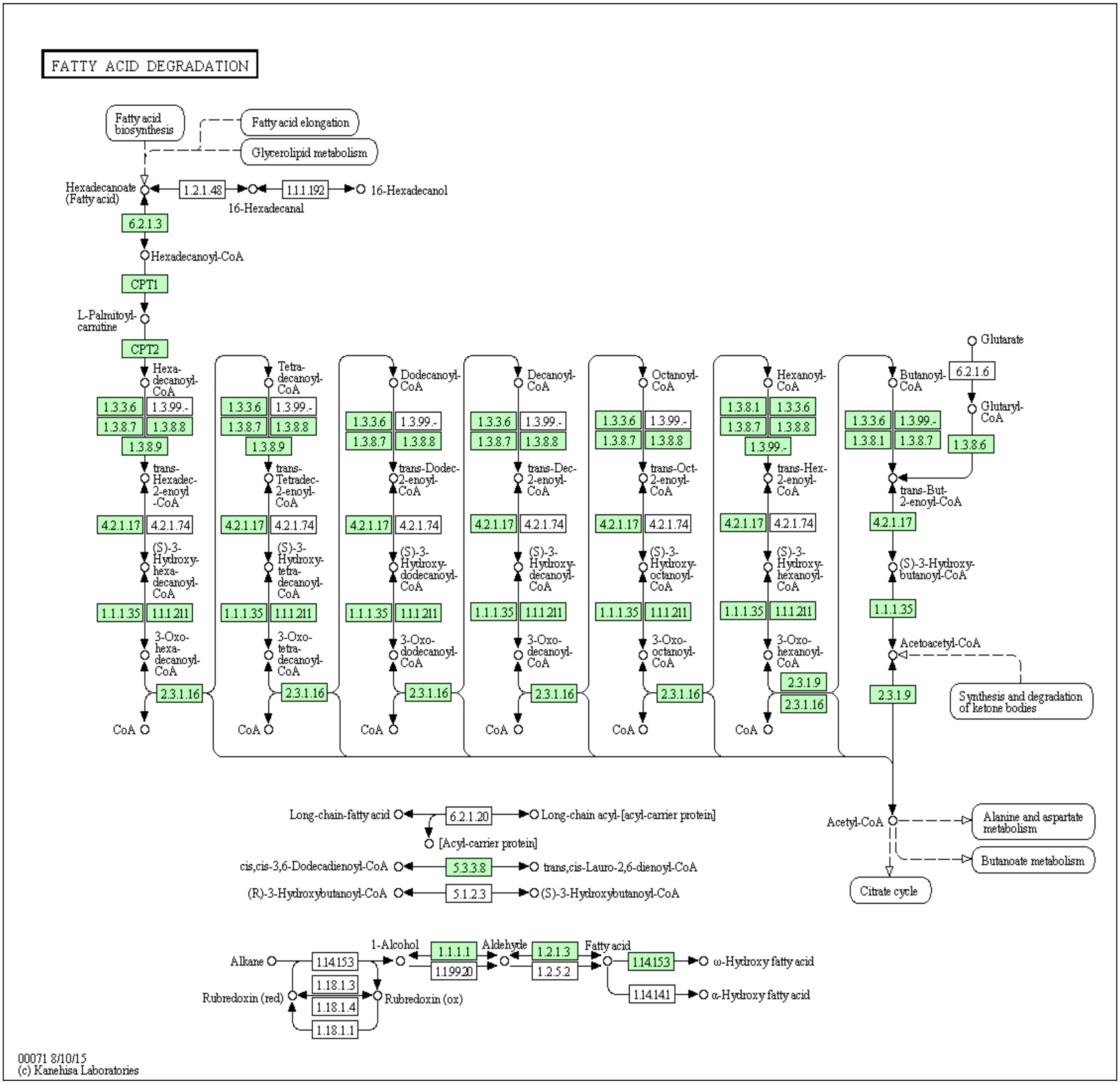
FATTY ACID DEGRADATION
Fatty acid biosynthesis
Fatty acid elongation
Glycerolipid metabolism
Hexadecanoate (Fatty acid)
1.2.1.48
1.1.1.192
16-Hexadecanol
16-Hexadecanal
6.2.1.3
Hexadecanoyl-CoA
CPT1
L-Palmitoyl-carnitine
CPT2
Hexa-decanoyl-CoA
Tetra-decanoyl-CoA
Dodecanoyl-CoA
Decanoyl-CoA
Octanoyl-CoA
Hexanoyl-CoA
Butanoyl-CoA
Glutarate
6.2.1.6
Glutaryl-CoA
1.3.8.6
1.3.3.6
1.3.99.-
1.3.8.7
1.3.8.8
1.3.8.9
1.3.8.1
trans-Hexadec-2-enoyl-CoA
trans-Tetradec-2-enoyl-CoA
trans-Dodec-2-enoyl-CoA
trans-Dec-2-enoyl-CoA
trans-Oct-2-enoyl-CoA
trans-Hex-2-enoyl-CoA
trans-But-2-enoyl-CoA
4.2.1.17
4.2.1.74
(S)-3-Hydroxy-hexa-decanoyl-CoA
(S)-3-Hydroxy-tetra-decanoyl-CoA
(S)-3-Hydroxy-dodecanoyl-CoA
(S)-3-Hydroxy-decanoyl-CoA
(S)-3-Hydroxy-octanoyl-CoA
(S)-3-Hydroxy-hexanoyl-CoA
(S)-3-Hydroxy-butanoyl-CoA
1.1.1.35
1.1.1.211
3-Oxo-hexa-decanoyl-CoA
3-Oxo-tetra-decanoyl-CoA
3-Oxo-dodecanoyl-CoA
3-Oxo-decanoyl-CoA
3-Oxo-octanoyl-CoA
3-Oxo-hexanoyl-CoA
Acetoacetyl-CoA
2.3.1.16
2.3.1.9
CoA
Synthesis and degradation of ketone bodies
Acetyl-CoA
Alanine and aspartate metabolism
Butanoate metabolism
Citrate cycle
Long-chain-fatty acid
6.2.1.20
Long-chain acyl-[acyl-carrier protein]
[Acyl-carrier protein]
cis,cis-3,6-Dodecadienoyl-CoA
5.3.3.8
trans,cis-Lauro-2,6-dienoyl-CoA
(R)-3-Hydroxybutanoyl-CoA
5.1.2.3
(S)-3-Hydroxybutanoyl-CoA
Alkane
1.14.15.3
1-Alcohol
1.1.1.1
1.1.99.20
Aldehyde
1.2.1.3
1.2.5.2
Fatty acid
1.14.15.3
ω-Hydroxy fatty acid
1.14.14.1
α-Hydroxy fatty acid
1.18.1.3
1.18.1.4
1.18.1.1
Rubredoxin (red)
Rubredoxin (ox)
00071 8/10/15
(c) Kanehisa Laboratories

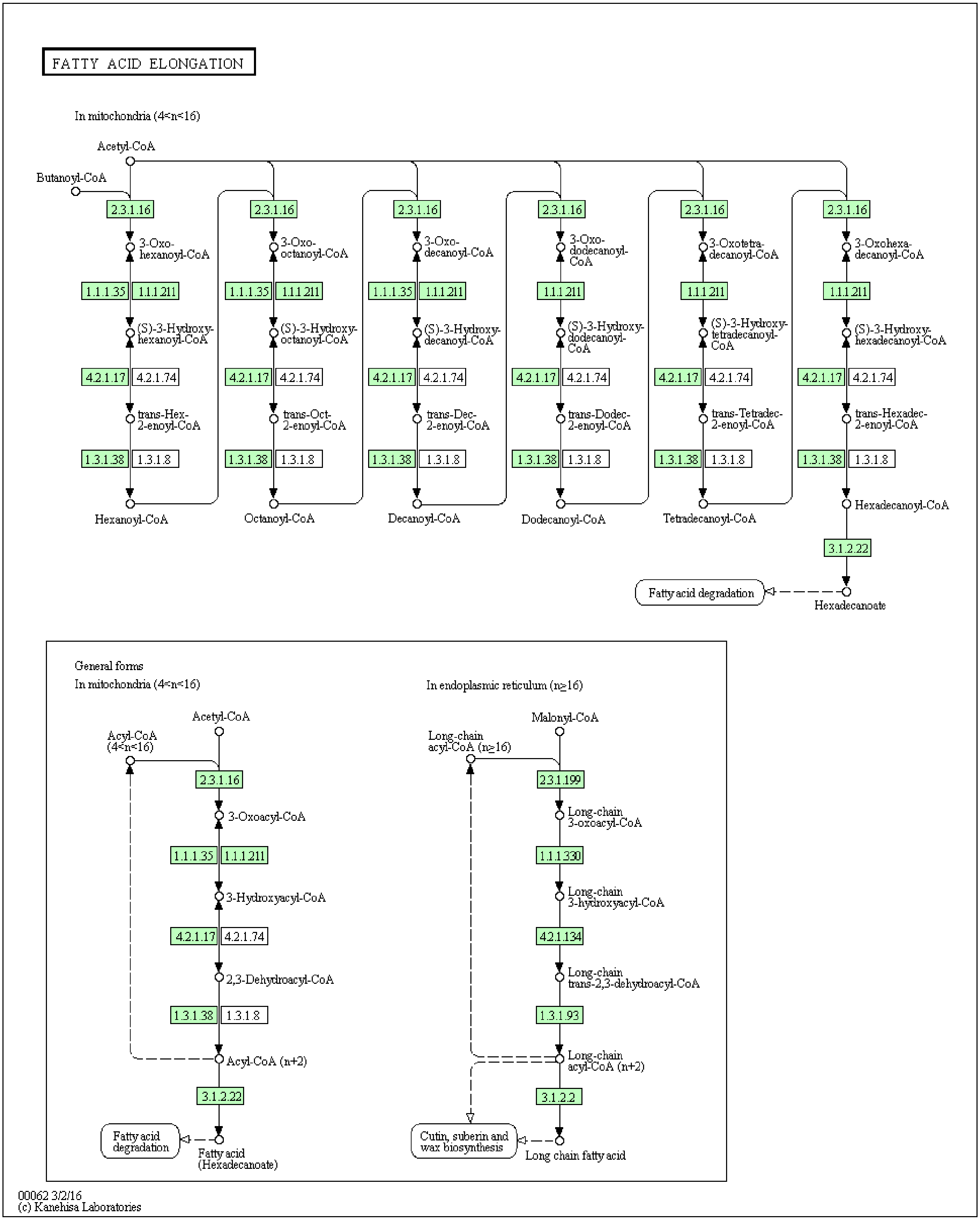
FATTY ACID ELONGATION
In mitochondria (4<n<16)
Acetyl-CoA
Butanoyl-CoA
2.3.1.16
3-Oxo-hexanoyl-CoA
1.1.1.35
1.1.1.211
(S)-3-Hydroxy-hexanoyl-CoA
4.2.1.17
4.2.1.74
trans-Hex-2-enoyl-CoA
1.3.1.38
1.3.1.8
Hexanoyl-CoA
3-Oxo-octanoyl-CoA
(S)-3-Hydroxy-octanoyl-CoA
trans-Oct-2-enoyl-CoA
Octanoyl-CoA
3-Oxo-decanoyl-CoA
(S)-3-Hydroxy-decanoyl-CoA
trans-Dec-2-enoyl-CoA
Decanoyl-CoA
3-Oxo-dodecanoyl-CoA
(S)-3-Hydroxy-dodecanoyl-CoA
trans-Dodec-2-enoyl-CoA
Dodecanoyl-CoA
3-Oxotetra-decanoyl-CoA
(S)-3-Hydroxy-tetradecanoyl-CoA
trans-Tetradec-2-enoyl-CoA
Tetradecanoyl-CoA
3-Oxohexa-decanoyl-CoA
(S)-3-Hydroxy-hexadecanoyl-CoA
trans-Hexadec-2-enoyl-CoA
Hexadecanoyl-CoA
3.1.2.22
Fatty acid degradation
Hexadecanoate
General forms
In mitochondria (4<n<16)
Acyl-CoA (4<n<16)
3-Oxoacyl-CoA
3-Hydroxyacyl-CoA
2,3-Dehydroacyl-CoA
Acyl-CoA (n+2)
Fatty acid degradation
Fatty acid (Hexadecanoate)
In endoplasmic reticulum (n≥16)
Malonyl-CoA
Long-chain acyl-CoA (n≥16)
2.3.1.199
Long-chain 3-oxoacyl-CoA
1.1.1.330
Long-chain 3-hydroxyacyl-CoA
4.2.1.134
Long-chain trans-2,3-dehydroacyl-CoA
1.3.1.93
Long-chain acyl-CoA (n+2)
3.1.2.2
Cutin, suberin and wax biosynthesis
Long chain fatty acid
00062 3/2/16
(c) Kanehisa Laboratories
